# Mammalian eIF4E2-GSK3β maintains basal phosphorylation of p53 to resist senescence under hypoxia

**DOI:** 10.1038/s41419-022-04897-4

**Published:** 2022-05-14

**Authors:** Lei Sun, He Yang, Dong He, Jian Chen, Zhiqiang Dong, Shaoxiang Luo, Huiting Liang, Yu Cao, Bingcheng Cai, Min Zhang

**Affiliations:** 1 College of Life Science and Technology, College of Biomedicine and Health, Huazhong Agricultural University, Wuhan, 430070 China; 2 Hubei Cancer Hospital (HBCH), Wuhan, 430079 China; 3 Wuhan Huamei Biotech Co., Ltd, Wuhan, 430079 China

**Keywords:** Kinases, Phosphorylation, Cancer microenvironment

## Abstract

Hypoxia modulates senescence, but their physiological link remains unclear. Here, we found that eIF4E2, a hypoxia-activated translation initiation factor, interacted with GSK3β to maintain phosphorylation of p53, thus resisting senescence under hypoxia. RNA-binding protein RBM38 interacted with eIF4E to inhibit the translation of p53, but GSK3β-mediated Ser195 phosphorylation disrupted the RBM38-eIF4E interaction. Through investigation of RBM38 phosphorylation, we found that the eIF4E2-GSK3β pathway specifically regulated proline-directed serine/threonine phosphorylation (S/T-P). Importantly, peptides e2-I or G3-I that blocking eIF4E2-GSK3β interaction can inhibit the basal S/T-P phosphorylation of p53 at multiple sites, therby inducing senescence through transcriptional inhibition. Additionally, a nanobody was screened via the domain where eIF4E2 bound to GSK3β, and this nanobody inhibited S/T-P phosphorylation to promote senescence. Furthermore, hypoxia inhibited eIF4E2-GSK3β pathway by mediating S-Nitrosylation of GSK3β. Blocking eIF4E2-GSK3β interaction promoted liver senescence under hypoxia, thus leading to liver fibrosis, eventually accelerating N, N-diethylnitrosamine (DEN)-induced tumorigenesis. Interestingly, eIF4E2 isoforms with GSK3β-binding motif exclusively exist in mammals, which protect zebrafish heart against hypoxia. Together, this study reveals a mammalian eIF4E2-GSK3β pathway that prevents senescence by maintaining basal S/T-P phosphorylation of p53, which underlies hypoxia adaptation of tissues.

## Introduction

Hypoxia plays a pivotal role in the pathogenesis of multiple human diseases [1]. Hypoxia-inducible factors (HIFs) mediates cellular adaptation to hypoxia by transcriptionally inducing a robust set of genes [1]. Adaptive protein synthesis is an alternative mechanism underlying hypoxia adaptation [2]. Hypoxia represses eukaryotic translation initiation factor 4E (eIF4E)-mediated translation, while its homolog eIF4E2 is activated and served as a translation initiation factor during hypoxia [3–5]. eIF4E2 specifically regulates translation by interacting with RNA-binding proteins [5, 6].

Senescence is a stable cell-cycle arrest which limits tissue damage and tumorigenesis by excluding damaged cells [7, 8]. However, persistent senescence can be detrimental to tissue homeostasis through senescence-associated secretory phenotype (SASP) [9]. Various cellular stresses promote senescence characterized by senescence-associated β-galactosidase activity (SA-β-gal) [7, 8]. Hypoxia averts stress-induced senescence in cultured cells [10, 11]. However, hypoxia can negatively or positively influence senescence in different physiological context, and the related mechanisms are poorly understood [12, 13].

Glycogen synthase kinase-3β (GSK3β), as a serine/threonine kinase, is highly active in resting cells and at the center of cellular signaling, which plays a key role in several physiological processes under hypoxia [14, 15]. GSK3β appears to both promote and oppose senescence under different circumstances [16–18]. GSK3β prefers primed substrates that require pre-phosphorylation (primed motif), but it is also considered as a proline-directed protein kinase [14, 19]. The mechanism by which these two kinase activities of GSK3β are distinguished is still unknown.

The p53 transcription factor mediates senescence, which contributes to tumor suppression [20, 21]. However, physiological p53 protects tissues from senescence [22, 23]. p53 is activated through its phosphorylation under multiple stresses. For example, genotoxic stress promotes the phosphorylation of p53-Ser46 [24], and non-genotoxic stress activates the phosphorylation of p53-Thr81 [25]. Notably, DNA damage specifically induces p53 phosphorylation at multiple Ser/Thr-Pro sites [26]. GSK3β mediates proline-directed phosphorylation of p53 [27], but its role remains unknown.

The RNA-binding protein RBM38 inhibits p53 translation by interacting with eIF4E that prevents eIF4E from binding to p53 mRNA [28]. GSK3β-mediated serine 195 (-Pro196) phosphorylation of RBM38 activates p53 translation by disrupting the RBM38-eIF4E interaction [29]. In this study, we found that RBM38 directly interacted with eIF4E2. The interrogation of RBM38 revealed the eIF4E2-GSK3β pathway that maintains the proline-directed phosphorylation of p53 at multiple serine/threonine sites (S/T-P), which resisting senescence.

## Materials and methods

### Reagents and plasmids

The used antibodies, other supplies, the cloning strategy, and primers used were listed in experimental procedures or supplementary materials.

### Cell culture, Transfection, and RNA Interference

HCT116, p53-null HCT116, eIF4E2-KO HCT116, H1299, MCF7, 293 T were cultured in DMEM (Gibco), A549 cells were cultured in RPMI medium 1640 (Hyclone). All media was supplemented with 10% fetal bovine serum (Hyclone), 100 units/ml penicillin, 100 µg/ml streptomycin. All cell lines were cultivated at 37 °C in 5% CO_2_ humidity. Plasmid or small interfering RNAs (siRNA) were transfected into cells according to Thermo Fisher protocol (L3000015, Lipofectamine 3000 Reagent). The Small interfering RNAs (siRNA) targeting eIF4E2#1 (5′ CUC ACA CCG ACA GCA UCA A dTdT 3′), siRNA targeting eIF4E2#2 (5′ CAC AGA GCU AUG AAC AGA AUA dTdT 3′) were used.

### Phosphoproteomic analysis using iTRAQ LC-MS/MS

HCT116 were treated with 5 μM e2-I or the scrambled peptide for 24 h and whole cell protein lysates were collected. The cell lysates were reduced with 10 mM DTT at 56 °C for 30 min, alkylated with 50 mM iodoacetamide (IAM) at room temperature for 30 min in the dark. The proteolytic solution was subjected to phosphopeptides enrichment using an Immobilized Metal Affinity Chromatography method [30]. Then, the peptides were labeled with iTRAQ Reagent-8 plex Multiplex Kit (AB Sciex U.K. Limited) according to the manufacturer’s protocol. Samples were iTRAQ labeled as following: ctrl_1,113; e2I_1, 114; ctrl_2, 115; e2I_2, 116. The enriched phosphopeptides were then dissolved in 2% acetonitrile/0.1% formic acid and analyzed using TripleTOF 5600+ mass spectrometer coupled with the Eksigent nanoLC System (AB SCIEX, USA). The original MS/MS file data were submitted to ProteinPilot Software v4.5 for data analysis. For protein identification, the Paragon algorithm which was integrated into ProteinPilot was employed against uniprot/swissprot-Homo database for database searching [31]. And proteins with a fold change larger than 1.5 and *p*-value < 0.05 were considered to be significantly differentially expressed. The Motif-x algorithm (http://motifx.med.harvard.edu) was used to extract motifs, and the significance threshold was set to *P* < 1e^−6^.

### RNA-sequencing, Senescence-associated β-galactosidase (SA-β-Gal) assay

The methods were described in experimental procedures or supplementary materials.

### Statistical analysis

All quantitative data were shown as mean ± S.D. The difference between two groups of variables was compared by the two-tailed, paired or unpaired Student’s *t*-test. For comparisons of more than two groups, one-way analysis of variance (ANOVA) was employed for normal distributions. *P*-value of <0.05 was considered as significant (*P < 0.05, **P < 0.01, and ***P < 0.001). Grayscale analysis of WB and quantification of cardiac or liver fibrosis or liver and cellular senescence were performed using ImageJ. All statistical analyses were performed using Graphpad Prism 8.0 for data analysis and imaging.

## Results

### eIF4E2 regulates GSK3β proline-directed kinase activity

Combining co-immunoprecipitation and mass spectrometry, we found that eIF4E2 might be a partner of RBM38. GST pull-down assays showed that eIF4E2 interacted with RBM38 (Supplementary Fig. 1A, B). The immunoprecipitation (IP) assays confirmed eIF4E2-RBM38 endogenous interaction (Supplementary Fig. 1 C, D). Unexpectedly, knockdown of eIF4E2 significantly down-regulated

**Fig. 1:**
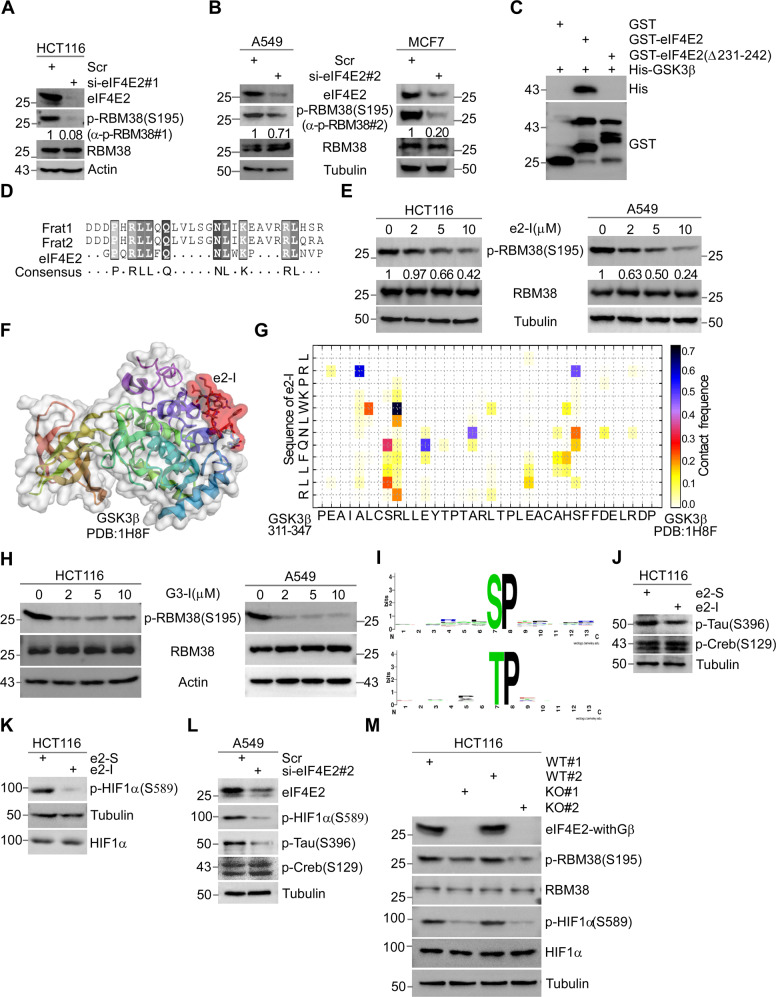
eIF4E2 regulates GSK3β proline-directed kinase activity. **A**, **B** Depletion of eIF4E2 inhibits the phosphorylation of RBM38-Ser195. eIF4E2 siRNA#1 was transfected into HCT116 cells (**A**) or siRNA#2 was transfected into A549 or MCF7 cells (**B**) for 72 h, followed by WB with indicated antibodies. Antibody p-RBM38#1 or p-RBM38#2 was used to detect p-RBM38(Ser195) as indicated. **C** Identifying the GSK3β binding motif of eIF4E2 by GST pull-down assay. GSK3β directly interacts with eIF4E2, but not with eIF4E2 mutant lacking amino acid from 231 to 242 (Δ231–242). **D** The GSK3β binding motif of eIF4E2 or FRAT (Frat1 and Frat2) were aligned. **E** e2-I inhibits the phosphorylation of RBM38-Ser195. Cells were treated with different concentrations of e2-I (2, 5, 10 μM) or scrambled e2-S (10 μM) for 24 h, followed by WB. **F** CABS-dock showed the binding of e2-I to GSK3β. The crystallographic structure of GSK3β (PDB ID: 1H8F) was used and the interaction interface was highlighted in red. **G** The contact diagram of GSK3β with e2-I, proposed by CABS-dock web server. **H** G3-I inhibits the phosphorylation of RBM38-Ser195. **I** The most over-represented motif is proline-directed serine/threonine regulated by eIF4E2-GSK3β pathway. Phosphorylation motifs were extracted by using Motif-x algorithm and threshold for significance was set to *P* < 0.000001. **J, K** e2-I inhibits the proline-directed phosphorylation, including Tau-Ser396 (**J**) and HIF1α-Ser589 (**K**) but does not affect the phosphorylation of Creb-Ser129 that is a serine site within priming motif. **L** Depletion of eIF4E2 inhibits the Tau-Ser396 and HIF1α-Ser589. eIF4E2 siRNA#2 was transfected into A549 cells for 72 h, followed by WB with indicated antibodies. **M** Knockout of eIF4E2 isoforms with GSK3β binding motif (eIF4E2-withGβ) inhibits the RBM38-Ser195 and HIF1α-Ser589. Lysis of eIF4E2-KO HCT116 (KO) and isogenic wild-type HCT116 (WT) cells, followed by WB with indicated antibodies.

RBM38-Ser195 phosphorylation in various cell lines with two specific RBM38-Ser195 phosphorylation antibodies used (Fig. 1A, B, Supplementary Fig. 1E) [29, 32].

GST pull-down assays showed a direct interaction between eIF4E2 and GSK3β (Supplementary Fig. 1F, G), and immunoprecipitation (IP) assays confirmed their interaction in vivo (Supplementary Fig. 1H, I). Mapping found that eIF4E2 mutant (Δ231–242), lacking amino acid from 231 to 242, did not bind to GSK3β (Fig. 1C). Coincidentally, this absent sequence (231–242 of eIF4E2) was highly homologous to a known GSK3β-binding motif of FRAT (Fig. 1D) [33]. Corresponding to this sequence, peptide e2-I fused with cell-penetrating peptide was synthesized with scrambled e2-S as a control. As expected, e2-I inhibited the eIF4E2-GSK3β interaction in GST pull-down assay (Supplementary Fig. 1J). Importantly, e2-I inhibited the phosphorylation of RBM38-Ser195 in a dose-dependent manner in various cell lines, but did not affect the protein expression of RBM38 (Fig. 1E). As a control, e2-S had effect on neither (Supplementary Fig. 1K). of note, the Ser195 was a proline-directed serine site.

Protein-peptide docking showed that e2-I bound to GSK3β, and a contact map indicated the potential eIF4E2-binding motif of GSK3β (Fig. 1F, G) [34]. GST pull-down assay showed that eIF4E2 interacted with GSK3β, but not with GSK3β mutant (Δ314–329) (Supplementary Fig. 1L). Peptide G3-I was synthesized corresponding to this sequence 314–329 of GSK3β. G3-I inhibited the eIF4E2-GSK3β interaction (Supplementary Fig. 1M), and significantly inhibited the phosphorylation of RBM38-Ser195 in various cell lines (Fig. 1H).

After blocking eIF4E2-GSK3β interaction by using e2-I, we performed iTRAQ-based quantitative phosphoproteomic analysis. Totally, 1882 altered phosphosites were identified from 1059 phosphoproteins. The proline-directed serine/threonine (S/T-P or S/T-X-P, X is any amino acid) was the most representative motif targeted by the eIF4E2-GSK3β pathway (Fig. 1I, Supplementary Fig. 1N) [35]. In contrast, no sites with primed motif were identified. For verification, e2-I inhibited the phosphorylation of Tau-Ser396 (followed by Pro397) identified in the phosphoproteomics, but it had no effect on Creb S129 phosphorylation, a known GSK3β-targeting site with primed motif (Fig. 1J) [36, 37]. In addition, e2-I inhibited phosphorylation of HIF1α-Ser589 identified in the phosphoproteomics (Fig. 1K) [38]. In vitro kinase assay showed that eIF4E2 further enhanced GSK3β-mediated phosphorylation of HIF1α-Ser589 (Supplementary Fig. 1O). Consistently, knockdown of eIF4E2 inhibited the proline-directed phosphorylation of Tau-Ser396 or HIF1α-Ser589 (Fig. 1L).

The eIF4E2 in human has seven isoforms with different termini, of which isoform A, E, and G have the GSK3β-binding motif (designated as eIF4E2-withGβ) (Supplementary Fig. 1P). Knockout cell line (eIF4E2-KO HCT116) was generated by CRISPR/Cas9 technology specially targeting the GSK3β-binding motif (Supplementary Fig. 1Q). Importantly, the level of phosphorylation of RBM38-Ser195 or HIF1α-Ser589 was decreased in eIF4E2-KO HCT116, compared with that in isogeneic HCT116 (Fig. 1M). Together, our results indicated that eIF4E2-GSK3β specifically regulated proline-directed phosphorylation.

### eIF4E2-GSK3β maintains p53 phosphorylation at multiple S/T-P sites

**Fig. 2:**
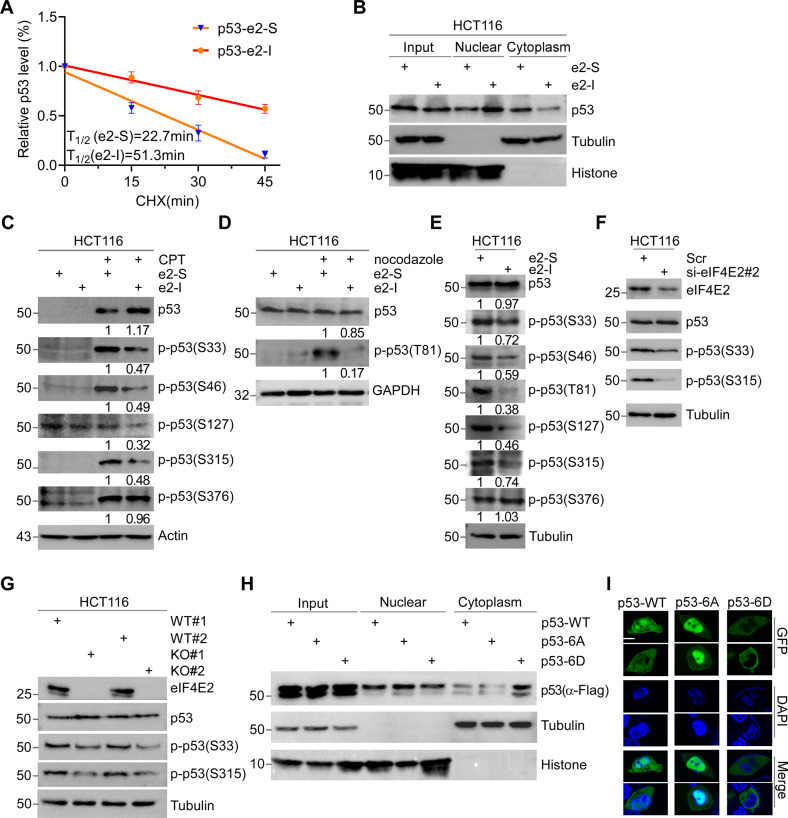
eIF4E2-GSK3β maintains p53 phosphorylation at multiple S/T-P sites. **A** HCT116 cells were treated with e2-I for 24 h followed by treatment with cycloheximide (CHX) for the indicated times. The intensity of p53 expression for each time point was quantified by grayscale analysis and plotted against time. **B** e2-I promotes nuclear localization of p53. Cells were treated with 5 μM e2-I or scrambled e2-S for 24 h, then the nuclear fraction was separated from the cytoplasmic fraction, followed by WB with antibodies against p53, Tubulin (markers for cytoplasmic), and Histone (markers for nuclear). **C** e2-I inhibits the CPT-induced phosphorylation of p53 at multi-S-P. Cells were treated with peptide as in (**B**), along with mock-treated or treated with 200 nM CPT for 24 h, followed by WB. **D** e2-I inhibits the nocodazole-induced phosphorylation of p53-Thr81. The experiment was done as described in (**C**), except that cell were mock-treated or treated with nocodazole (50 ng/ml). **E** e2-I inhibits multi-S/T-P phosphorylation of p53 at basal conditions. **F** Depletion of eIF4E2 inhibits the phosphorylation of p53-Ser33 and p53-Ser315. eIF4E2 siRNA#2 was transfected into HCT116 cells for 72 h, followed by WB with indicated antibodies. **G** Knockout of eIF4E2 inhibits the phosphorylation of p53-Ser33 and p53-Ser315. Lysis of eIF4E2-KO HCT116 (KO) and isogenic wild-type HCT116 (WT) cells, followed by WB with indicated antibodies. **H** Mutant p53-6A is preferentially located in the nuclear. Vectors expressing FLAG tagged p53, p53-6A or p53-6D, were transfected into p53-null HCT116 cells for 48 h. The nuclear and cytoplasmic fractions were separated, followed by WB. **I** Green fluorescent signals showed that mutant p53-6A is preferentially located in nuclear. Vectors expressing GFP-fused p53, p53-6A or p53-6D, were transfected into p53-null HCT116 cells for 24 h. GFP fluorescence and DAPI staining were observed using confocal microscopy. Scale bars, 5 μm.

We speculated that eIF4E2-GSK3β pathway might regulate p53 translation through RBM38-Ser195 phosphorylation [29]. By L-azidohomoalaine (AHA) labeling [39], we showed that the newly synthesized p53 was decreased upon e2-I treatment (Supplementary Fig. 2A, left panel). However, e2-I had little effect on the whole protein expression of p53 (Supplementary Fig. 2A, right panel). This might be explained by the fact that e2-I extended the half-life of p53 protein [40] (Fig. 2A, Supplementary Fig. 2B). Consistently, e2-I inhibited the expression of cytoplasmic p53, but activated nuclear p53 expression (Fig. 2B).

eIF4E2-GSK3β pathway might regulate p53 stability through its S/T-P phosphorylation (Supplementary Fig. 2C). After HCT116 cells were treated with e2-I under basal or stressed conditions, we found that camptothecin-induced DNA damage activated p53 phosphorylation at Ser33, Ser46, and Ser315 sites, but e2-I inhibited this activation (Fig. 2C) [41]. Additionally, e2-I inhibited the activation of p53-Thr81 phosphorylation induced by microtubule inhibitors nocodazole (Fig. 2D) [42]. p53-Ser127 phosphorylation did not respond to stress [43], and Thr150 phosphorylation was undetectable in this study. Notably, basal S/T-P phosphorylation of p53 was suppressed by e2-I (Fig. 2E). For comparison, Ser376 (a known GSK3β-targeting site with no proline) phosphorylation of p53 exhibited no change (Fig. 2C-E) [44]. Similar to e2-I, G3-I inhibited multiple S/T-P phosphorylation of p53 under both basal and stressed conditions (Supplementary Fig. 2D–F). Consistently, knockdown of eIF4E2 or knockout of eIF4E2-withGβ down-regulated the proline-directed phosphorylation of p53 (Fig. 2F, G).

S/T-P dephosphorylation might suppress cytoplasmic degradation of p53 by regulating its cellular distribution. We generated vectors expressing p53 mutant 6 A (S/T-P mutated to A-P, A is alanine) and 6D (D is aspartic acid), for mimicking unphosphorylated and phosphorylated p53 respectively. We found that p53-6A was preferentially expressed in nucleus, whereas p53-6D in cytoplasm (Fig. 2H). GFP fluorescence fused with p53 showed similar cellular localization of 6 A and 6D (Fig. 2I). Overall, our results suggested that eIF4E2-GSK3β pathway maintained the multiple S/T-P phosphorylation of p53, which regulates the cellular localization of p53, thus modulating its stability.

### Dephosphorylated p53 promotes senescence by repressing transcription

**Fig. 3:**
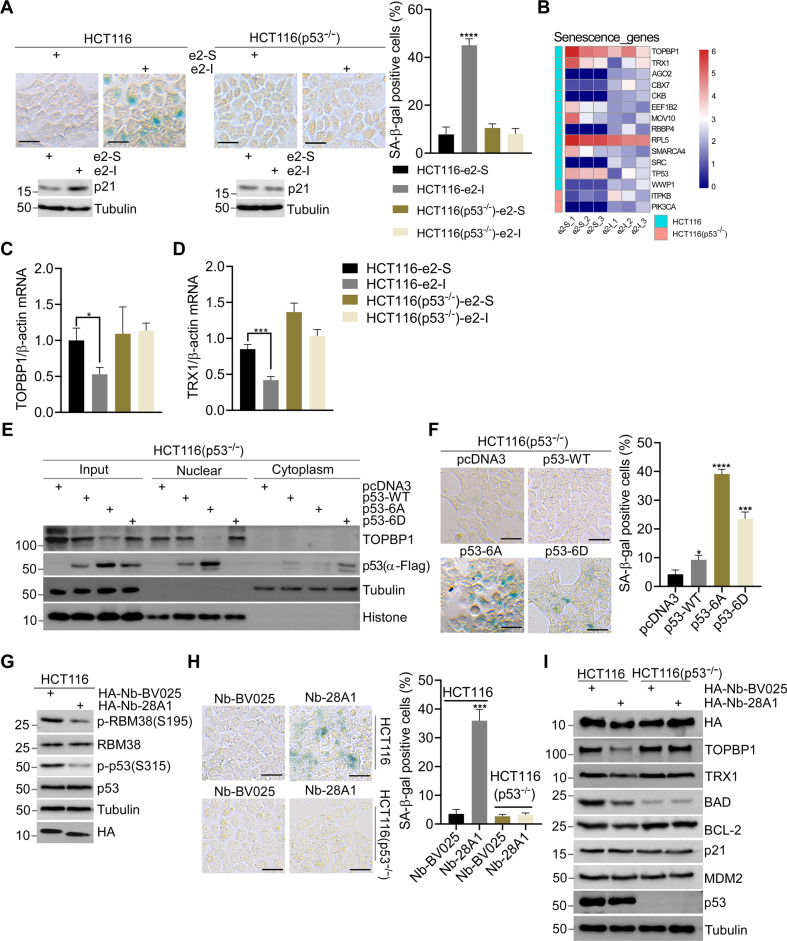
Dephosphorylated p53 promotes senescence by repressing transcription. **A** SA-β-Gal staining of HCT116 and p53-null HCT116 cells treated with 5 μM e2-I or scrambled e2-S for 96 h (upper panel). And quantitative analysis percentages of SA-β-Gal positive cells (right panel *n* = 3). Followed by WB with indicated antibodies (lower panel). Scale bars, 25 μm. **B** Heat map showed the differential expression genes associated with senescence in HCT116 and p53-null HCT116 cells, respectively. Differential expression genes (DEGs) were identified upon e2-I treatment by RNA-Seq. **C, D** e2-I inhibits mRNA expression of TOPBP1 or TRX1 depending on p53. HCT116 or HCT116-p53 null cells were treated with 5 μM e2-I or scrambled e2-S for 24 h. Total RNA was extracted and real-time quantification reverse transcriptase polymerase chain reaction (QRT-PCR) was performed. **E** Mutant p53-6A suppresses the expression of TOPBP1. Vectors expressing FLAG tagged p53, p53-6A or p53-6D, were mock-transfected or transfected into p53-null HCT116 cells for 48 h. Then, the nuclear fraction was separated from the cytoplasmic fraction, followed by WB. **F** SA-β-Gal staining of p53-null HCT116 cells expressing FLAG tagged (p53-WT, p53-6A, p53-6D, and mock) for 96 h (left panel), and quantitative analysis of the percentages of SA-β-Gal positive cells (*n* = 3, right panel). Scale bars, 25 μm. **G** Expression of nanobody Nb-28A1 inhibits the phosphorylation of RBM38-Ser195 and p53-Ser315. Vector pcDNA3-HA-Nb-28A1 or control vector pcDNA3-HA-Nb-BV025 was transfected into HCT116 cells for 48 h, followed by WB with indicated antibodies. **H** Nanobody Nb-28A1 induced cell senescence depending on p53. SA-β-Gal staining of HCT116 or p53-null HCT116 cells expressing HA-Nb-28A1 or HA-Nb-BV025 for 96 h (left panel), and quantitative analysis percentages of SA-β-Gal positive cells (*n* = 3, right panel). Scale bars, 25 μm. **I** Expression of nanobody HA-Nb-28A1 for 24 h downregulates the expression of TOPBP1, TRX1, BCL2 or BAD depending on p53. Vector pcDNA3-HA-Nb-28A1 or control vector pcDNA3-HA-Nb-BV025 was transfected into HCT116 or p53-null HCT116 cells for 24 h, followed by WB with indicated antibodies.

By SA-β-Gal staining, we found that 96-h e2-I treatment promoted senescence in p53-wildtype cells, but not in p53-null cells (Fig. 3A, Supplementary Fig. 3A, upper panel). The 96-h e2-I treatment activated the expression of senescence marker p21 in p53-dependent manner (Fig. 3A, Supplementary Fig. 3A, lower panel). In addition, e2-I, rather than scrambled e2-S, inhibited the growth of HCT116 xenografts, (Supplementary Fig. 3B, C), but e2-I had no effect on p53-null HCT116 xenografts (Supplementary Fig. 3B, C). These results revealed that the effect of the eIF4E2-GSK3β pathway on senescence was p53-dependent.

We performed transcriptome analysis of p53-wildtype and p53-null HCT116 after treatment with e2-S and e2-I. GSEA assay showed that eIF4E2-GSK3β pathway was significantly associated with WNT signaling or neurodegenerative disease (Supplementary Fig. 3D, E), both of which are highly related to GSK3β [14]. By referring to database of senescence-genes, 13 differentially expressed genes (DEGs) associated with senescence were found in HCT116 upon e2-I treatment, while only two in p53-null HCT116 upon e2-I treatment (Fig. 3B). Quantitative RT-PCR confirmed that e2-I (24-h) inhibited TOPBP1 and TRX1 depending on p53 (Fig. 3C, D). The expression of p53-6A (48-h) inhibited the expression of TOPBP1 and TRX1, whereas p53-WT or mutant 6D did not (Fig. 3E, Supplementary Fig. 3F). The inhibition of Topoisomerase II binding protein 1 (TOPBP1) and thioredoxin-1 (TRX1) has been reported to promote senescence by elevating the expression of p21 [45, 46]. Consistently, we observed that 96-h p53-6A expression promoted senescence (Fig. 3F). However, p53-6D also promoted senescence, indicating that S/T-P phosphorylation of p53 might induces senescence, which might be attributed to stress conditions (Fig. 3F).

To further validated the transcription repression activity of the inhibition of eIF4E2-GSK3β pathway, more targets were checked according to the transcriptome results. The RT-PCR results showed that peptide e2-I treatment (24-h), as well as overexpression of p53-6A (24-h) inhibited the mRNA expression of HNRNPD, EEF2, GAMT, NEK9 (Supplementary Fig. 4A, B). The examination of more known transcriptional targets of p53 revealed that e2-I treatment (24-h) or overexpression of p53-6A (24-h) inhibited the mRNA expressions of BCL2 and BAD, both of which have effect on apoptosis with opposite effect [47] (Supplementary Fig. 4A, B). Importantly, Western blotting confirmed that 24-h e2-I treatment inhibited TOPBP1, TRX1, BCL2 or BAD in p53-dependent manner (Supplementary Fig. 4C), but not p21 and MDM2. These results suggested that the activation of p21 upon 96-h e2-I treatment is an indirect and late event responsible for the development of senescence. Consistently, we found that e2-I treatment (24-h) and expression of p53-6A (24-h) had no effect on either VEGF or GSN (Supplementary Fig. 4A, B), two known genes regulated by senescence [48, 49]. These data suggested that the transcriptional repression resulted from inhibition of eIF4E2-GSK3β pathway is a general and direct effect, which is a cause rather than a consequence of senescence.

Jaspar program predicted p53 responsive elements (REs) within the promoter of TOPBP1 and TRX1 (Supplementary Fig. 4D, E) [50]. CHIP assays confirmed that p53 bound to these promoter regions containing REs (Supplementary Fig. 4F, G). p53-6D exhibited a weak binding to TOPBP1 and TRX1 promoter. Luciferase reporter assays showed that p53-6A and p53-WT, rather than p53-6D, inhibited the activity of pGL3-TOPBP1 construct containing REs, but they did not affect pGL3-TOPBP1 (ΔREs) construct (Supplementary Fig. 5A, B). p53-6A, p53-WT, and p53-6D inhibited pGL3-TRX1 activity, of which p53-6A exhibited the largest inhibitory effect (Supplementary Fig. 5C, D).

Single-domain antibodies (nanobodies) targeting the interaction interface can be used to block protein-protein binding [51]. We isolated nanobodies to disrupt the eIF4E2-GSK3β interaction by screening a yeast surface-displayed library of synthetic nanobody sequences. GST pull-down assay demonstrated that Nb-28A1, one of our screened nanobodies, directly interacted with eIF4E2, but not with its mutant containing no GSK3β-binding domain (Supplementary Fig. 5E). eIF4E2 co-immunoprecipitated with HA-tagged nanobody Nb-28A1, but not with the control nanobody Nb-BV025 [52] (Supplementary Fig. 5F). Nb-28A1 did not recognize FRAT containing a similar GSK3β binding domain, confirming the high specificity of Nb-28A1 to eIF4E2 (Supplementary Fig. 5E, F). Nb-28A1 blocked the eIF4E2-GSK3β interaction in GST pull-down assay (Supplementary Fig. 5G). More importantly, expression of HA-Nb-28A1 (24-h) inhibited the phosphorylation of RBM38-Ser195 and p53-Ser315 (Fig. 3G). Expression of Nb-28A1 (96-h) promoted cellular senescence in HCT116 cells but not in p53-null HCT116 (Fig. 3H). Consistent with inhibitory effect of e2-I, expression of Nb-28A1 (24-h) inhibited the expression of TOPBP1, TRX1, BCL2 and BAD in p53-dependent manner (Fig. 3I), and mRNA expression of HNRNPD, EEF2, GAMT, NEK9, BCL2, BAD, but affected neither VEGF nor GSN (Supplementary Fig. 5H).

### Hypoxia inhibits the eIF4E2-GSK3β pathway

**Fig. 4:**
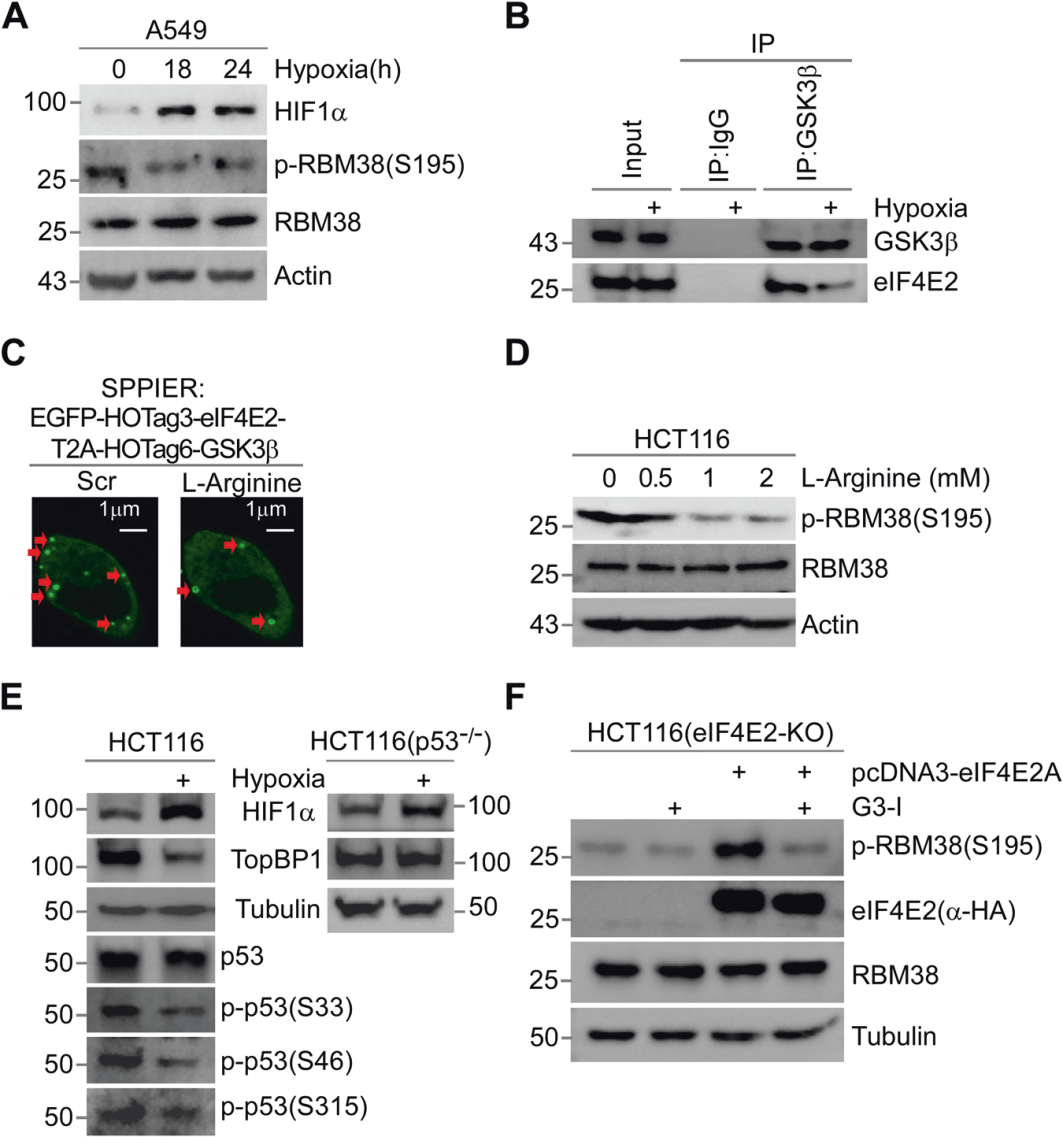
Hypoxia inhibits the eIF4E2-GSK3β pathway. **A** Hypoxia inhibits the phosphorylation of RBM38-Ser195. A549 cells were exposure to hypoxia (1% O_2_) for 18, 24 h, or normoxia for 24 h, followed by WB. **B** Hypoxia inhibits eIF4E2-GSK3β binding. Cells were exposure to normal or hypoxia (1% O_2_) condition for 24 h. Then, cell extracts were subjected to Co-IP with anti-GSK3β antibody or IgG, followed by WB. **C** SPPIER assay showed L-Arginine inhibits eIF4E2-GSK3β interaction. Cells transiently expressed EGFP-eIF4E2-HOTag3-T2A-GSK3β-HOTag6, and then 2mM L-Arginine was added to the cells for 1 h. Scale bars, 1 μm. **D** L-Arginine inhibits the phosphorylation of RBM38- Ser195. Cells were mock-treated or treated with different concentrations of L-Arginine (0.5, 1, 2 mM) for 24 h and subjected to WB. **E** Hypoxia inhibits the phosphorylation of p53-Ser33, Ser46, Ser315 and the expression of TOPBP1. HCT116 (left) or p53-null HCT116 (right) cells were exposure to normoxia or hypoxia (1% O_2_) condition for 18 h, followed by WB with indicated antibodies. **F** Expression of eIF4E2 isoform A activates the phosphorylation of Rbm38-Ser195 in eIF4E2-KO HCT116 cells. Vectors expressing eIF4E2 isoform A with HA-tag were mock-transfected or transfected into eIF4E2-KO HCT116 cells for 48 h respectively, along with treatment with 5 μM G3-I or scramble peptide as indicated for 24 h, followed by WB.

Considering that eIF4E2 was activated under hypoxia, we inferred that eIF4E2-GSK3β pathway might play a role under hypoxia. We observed that hypoxia significantly inhibited phosphorylation of RBM38-Ser195 in different cell lines (Fig. 4A, Supplementary Fig. 6A). eIF4E2 was detected in the anti-GSK3β immune complex under normal conditions, whereas less eIF4E2 was detected under hypoxia (Fig. 4B). The phase separation-based protein interaction reporter (SSPIER) analysis indicated that eIF4E2-GSK3β interaction led to the phase separation, thus prompting the formation of EGFP droplets, but 2-h hypoxia exposure demolished EGFP droplets (Supplementary Fig. 6B) [53, 54].

The S-Nitrosylation at cysteine can inhibit proline-directed kinase activity of GSK3β [19]. Interestingly, Cys317 was located in the domain where GSK3β bound to eIF4E2. L-Arginine is used as a substrate for the production of nitric oxide to induce S-Nitrosylation [55]. The SSPIER analysis revealed that L-Arginine inhibited eIF4E2-GSK3β interaction (Fig. 4C). Western blot showed that L-Arginine inhibited phosphorylation of RBM38-Ser195 (Fig. 4D), and that L-NAME treatment partially restored the phosphorylation of RBM38-Ser195 under hypoxia (Supplementary Fig. 6C). *N*^G^-nitro-L-Arginine methylester (L-NAME), as a NOS inhibitor, can inhibit S-Nitrosylation by blocking NO generation [56]. Furtherly, we found that hypoxia inhibited S/T-P phosphorylation of p53 at Ser33, Ser46, or Ser315, and inhibited TOPBP1 expression depending on p53 (Fig. 4E).

To determine the role of eIF4E2-GSK3β pathway under hypoxia, overexpressed eIF4E2 isoform A was introduced into eIF4E2-KO HCT116 cells. We found that the expression of eIF4E2 isoform A activated RBM38-Ser195 phosphorylation, which can be blocked by peptide G3-I (Fig. 4F). It takes a long period of time for hypoxia to induce senescence, during which hypoxia inhibited the growth of eIF4E2-KO HCT116, making senescence difficult to be detected. However, expression of eIF4E2 isoform A preserved the normal growth of eIF4E2-KO HCT116 under hypoxia (Supplementary Fig. 6D, E). These results suggested that hypoxia inhibited eIF4E2-GSK3β activity by inducing S-Nitrosylation of GSK3β.

### Blocking eIF4E2-GSK3β interaction promotes liver senescence under hypoxia

**Fig. 5:**
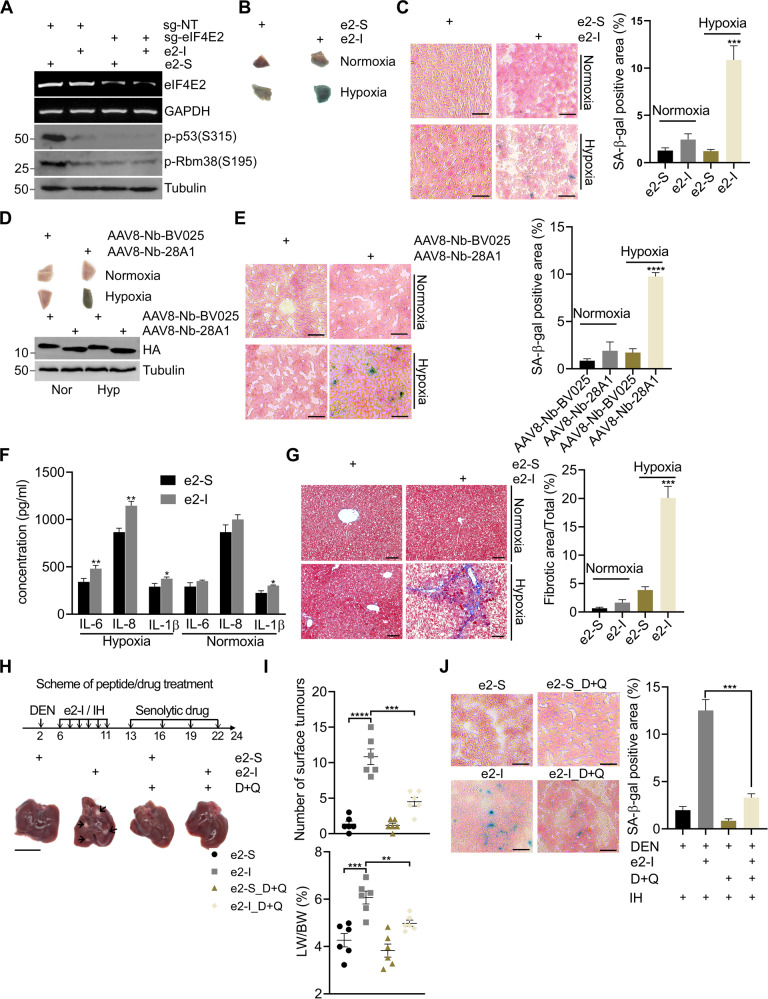
Blocking eIF4E2-GSK3β interaction promotes liver senescence under hypoxia. **A** Plasmids expressing CasRx-GFP, sgRNAs targeting eIF4E2 (sg-eIF4E2) or non-targeting (NT) guide RNAs were delivered to mouse livers by hydrodynamic tail-vein injection. 96 hr later, GFP^+^ hepatocytes were sorted for the quantification of mRNA or protein expression levels. **B**, **C** e2-I promoted liver senescence under intermittent hypoxia (IH). After intraperitoneal injection of 35 mg/kg e2-I or scrambled e2-S, mice were housed at normoxia or physiological hypoxia (12% O_2_) conditions and on the 5th day, liver tissue blocks were stained for SA-β-Gal activity (**B**), then sectioned and counterstained with nuclear fast red (**C**, left panel), and SA-β-Gal staining results were quantified by the ratio of SA-β-Gal^+^ area to the total image area for three fields (**C**, *n* = 3, right panel). Scale bars, 25 μm. **D**, **E** Nanobody Nb-28A1 promoted liver senescence under intermittent hypoxia (IH). AAV8-Nb-28A1 or AAV8-Nb-BV025 (1×10^11^viral genomes in 100 μl saline) were intravenous injected. Two weeks later, mice were housed at normoxia or physiological hypoxia (12% O_2_) conditions and on the 5th day, liver tissue blocks were stained for SA-β-Gal activity (D, upper panel), equal amounts of liver tissue were subjected to WB for checking the expression of nanobody (**D**, lower panel). Then sectioned and counterstained with nuclear fast red (**E**, left panel), and SA-β-Gal staining results were quantified by the ratio of SA-β-Gal^+^ area to the total image area for three fields (*n* = 3) (**E**, right panel). Scale bars, 25 μm. **F** e2-I induced excessive senescence-associated secretory phenotype (SASP). Mice (6 weeks old) were injected intraperitoneally with 35 mg/kg e2-I or scrambled e2-S twice a week for 6 weeks. After each injection, the mice were exposed to physiological hypoxia (12% O_2_) conditions for 8 h. At 8 weeks, the levels of IL-6, IL-8 and IL-1β in serum of mice were detected by using ELISA kits. Data are presented as mean SD from each group (*n* = 3). **G** e2-I leads to liver fibrosis. The liver fibrosis of mice from experiment (**F**) was evaluated by Masson’s trichrome staining and representative results were shown (left panel). Scale bars, 50 μm. Quantified data of the fibrosis area (right panel). Statistical Significance was determined by one-way analysis of variance (*n* = 4 per group). **H**, **I** Scheme of e2-I/IH or senolytics drug (dasatinib and quercetin) administration of DEN-treated mice (upper panel). e2-I/IH treatment was performed as described in (**F**). Representative macroscopic photographs of livers (*n* = 6) indicate the hepatocellular carcinoma (HCCs) by arrowheads. Scale bar: 1 cm (H). Number of surface tumors and LW/BW ratios in mice liver at 24 weeks and shown in the scatter plot (*n* = 6) (**I**). **J** Representative SA-β-Gal staining of 24-week mouse liver sections from experiment (**H**) (left panel). Scale bar: 25 µm. SA-β-Gal staining results were quantified by the ratio of SA-β-Gal^+^ area to the total image area for three fields (*n* = 3) (right panel).

To explore the physiological role of the eIF4E2-GSK3β pathway, peptides were intraperitoneally injected to mice. We found that e2-I injection effectively inhibited the phosphorylation of RBM38-Ser195 in liver, compared with scrambled e2-S. We used CasRx system to validate this e2-I effect is reached through eIF4E2-GSK3β pathway [57]. After hydrodynamic injection with CasRx and sg-eIF4E2 mixture, eIF4E2 mRNA levels were decreased in GFP^+^ hepatocytes, along with the decreased phosphorylation of RBM38-Ser195 and p53-Ser315. However, e2-I had no further effect on their phosphorylation in eIF4E2 knockdown hepatocytes (Fig. 5A).

The intraperitoneal injection of e2-I strikingly reduced mice viability under physiological hypoxia (12% O_2_) with the decreased ambulation counts and rearing counts of mice (Supplementary Fig. 7A, B). However, the effect of e2-I on mice viability was negligible under normoxic conditions.

More importantly, under physiological hypoxia, e2-I induced liver senescence (Fig. 5B, C), and inhibited the phosphorylation of p53-Ser315 and the expression of TOPBP1 or TRX1 (Supplementary Fig. 7C). Immunohistochemistry (IHC) results showed that e2-I induced expression of p53 or p21 in liver under physiological hypoxia (Supplementary Fig. 7D). Impressively, expression of TOPBP1 mediated by adeno-associated virus (AAV)8 rescued the e2-I-induced liver senescence under physiological hypoxia (Supplementary Fig. 7E). Similarly, G3-I induced liver senescence under physiological hypoxia, which was bypassed by the expression of AAV8-TRX1 (Supplementary Fig. 7F). Consistently, AAV8-Nb-28A1 induced senescence under hypoxia, compared with control AAV8-Nb-BV025 (Fig. 5D, E).

To explored the pathological role of the eIF4E2-GSK3β pathway, chronic intermittent hypoxia (IH) condition was established [58]. We intraperitoneally injected e2-S/e2-I to mice followed by IH. Two months later, e2-I, rather than e2-S, induced senescence (Supplementary Fig. 8A, B) and excessive senescence-associated secretory phenotype (SASP), as shown by increased secretion of proinflammatory cytokines such as IL-6, IL-8 and IL-1β (Fig. 5F). Subsequently, e2-I promoted liver fibrosis under IH (Fig. 5G). Persistent senescence accompanied by excessive SASP might promote tumorigenesis. The neonatal mice were treated with diethylnitrosamine (DEN) to initiate tumorigenesis, and then treated with peptide (e2-S/e2-I), followed by IH. Since senolytics drugs (dasatinib and quercetin, D + Q) can significantly blunt liver tumor progression with few liver lesions through elimination of senescent cells [59], we treated mice with D + Q after peptide/IH treatment (Fig. 5H, upper panel). We found that as early as 24 weeks, hepatocellular carcinoma (HCC) developed upon e2-I/IH treatment (Fig. 5H, I) or G3-I/IH treatment (Supplementary Fig. 8C, D), but not upon scrambled peptides/IH treatment. Impressively, senolytics drug blunted tumor progression accelerated by e2-I/G3-I treatment under IH (Fig. 5H, I, Supplementary Fig. 8C, D). The possible reason might lie in that senolytics drug efficiently eliminated SA-β-Gal^+^ cells induced by peptide/IH treatment (Fig. 5J). In contrast, e2-I/G3-I treatment did not advance HCC under normoxic condition (Supplementary Fig. 8E). These results suggested the protective roles of eIF4E2-GSK3β in tissues under physiological hypoxia.

### Mammalian eIF4E2 protects heart of zebrafish

**Fig. 6:**
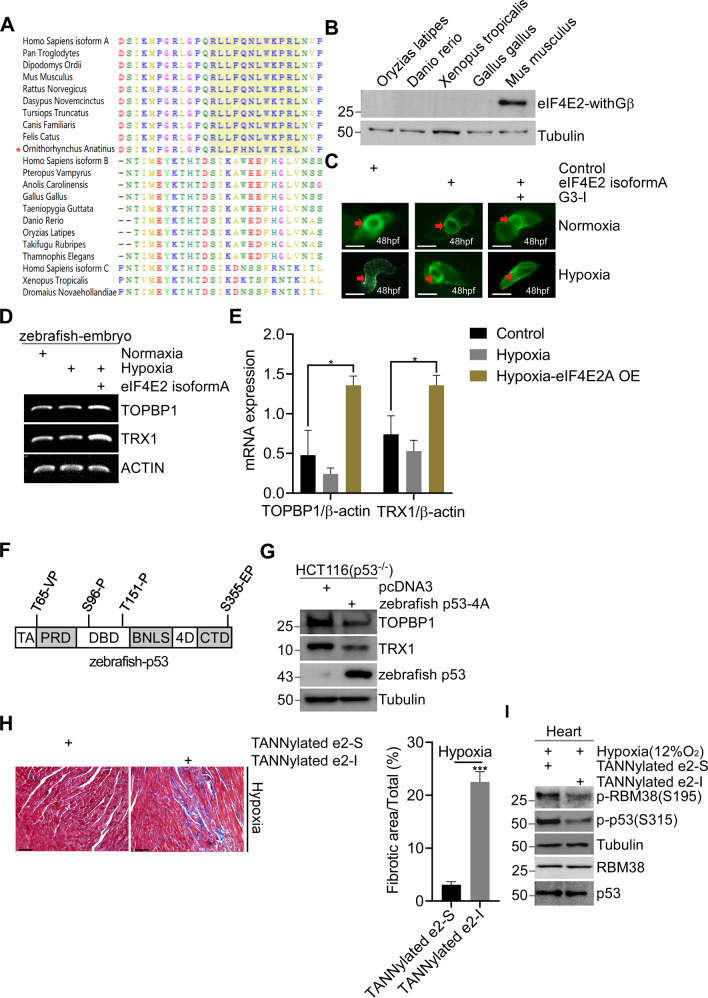
Mammalian eIF4E2 protects heart of zebrafish. **A** Sequence alignment of c-termini of eIF4E2 from major species, including mammals, reptiles, amphibians, fish, and birds. **B** WB detects the protein expression of eIF4E2-withGβ isoforms in different non-mammals. **C** Human eIF4E2 isoform A prevents disorder of heart looping of zebrafish. Vectors expressing eIF4E2 isoform A were mock-injected or injected into embryos (*n* = 5) at the 1-cell stage. 24 h later, embryos were exposure to hypoxic (1% O_2_) or normoxia for 24 h, and representative Tg (cmlc2: eGFP) heart were showed at 48 h. **D, E** Expression of eIF4E2 isoform A increases the mRNA expression of TOPBP1 or TRX1 in zebrafish embryo. Total RNA was extracted and reverse transcriptase polymerase chain reaction (RT-PCR) was performed (**D**). Real-time quantification reverse transcriptase polymerase chain reaction (QRT-PCR) was performed (**E**). **F** S/T-P sites of zebrafish p53 were indicated. **G** Zebrafish p53-4A mutant suppresses the expression of TOPBP1 or TRX1. Vectors expressing Zebrafish p53-4A, were mock-transfected or transfected into p53-null HCT116 cells for 48 h, followed by WB. **H** TANNylated e2-I or e2-S was intravenously injected to mice twice a week for 6 weeks, followed by intermittent hypoxia (IH) exposure (12% O_2_ for 8 h after injection). Cardiac fibrosis was evaluated by Masson’s trichrome staining and representative macroscopic photographs of heart tissue was showed (*n* = 4) (left panel). Quantified data of the fibrosis area. Statistical significance was determined by one-way analysis of variance (*n* = 4) (right panel). **I** Mice were intravenously injected with TANNylated e2-I or e2-S, and housed at physiological hypoxia (12% O_2_) conditions for 48 h. Then, heart tissues were analyzed by WB.

According to NCBI GenBank, eIF4E2 isoforms with GSK3β-binding motif (eIF4E2-withGβ) appears only in mammals (Fig. 6A). We further confirmed that non-mammalians such as Oryzias latipes, Danio rerio, Xenopus tropicalis and Gallus gallus did not express eIF4E2-withGβ isoforms (Fig. 6B). We speculated that eIF4E2-withGβ expression might exert protective role in non-mammalian by interacting with their own GSK3β since GSK3β is highly conserved. We introduced human eIF4E2 isoform A into zebrafish Tg (cmlc2: eGFP) embryos. The expression of eIF4E2 isoform A rescued abnormal heart loop caused by hypoxia stress (Fig. 6C), and G3-I hindered this rescue effect (Fig. 6C). RT-PCR results showed that eIF4E2 expression increased the expression of TOPBP1 and TRX1 (Fig. 6D, E). In addition, expression of zebrafish p53-4A (S/T-P to A-P) significantly inhibited their expression in p53-null HCT116 (Fig. 6F, G).

Recently, tannic acid modification (TANNylated) was established to deliver peptide to the heart [60]. After intravenous injection with TANNylated e2-I/e2-S to mice and IH treatment, we found that TANNylated e2-I rather than e2-S induced cardiac fibrosis (Fig. 6H). TANNylated e2-I inhibited the phosphorylation of RBM38-Ser193 and p53-Ser315 in heart under physiological hypoxia, indicating the conservative function of eIF4E2-GSK3β pathway in different tissues (Fig. 6I).

## Discussion

**Fig. 7:**
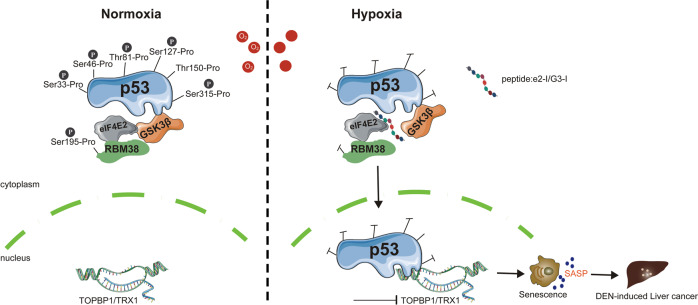
Model showing function of the eIF4E2-GSK3β. Left: eIF4E2-GSK3β maintains p53 phosphorylation at multiple S/T-P sites. Right: Peptides e2-I or G3-I blocking eIF4E2-GSK3β interaction can inhibit multiple S/T-P phosphorylation of p53, which subsequently transcriptional inhibiting the expression of TOPBP1 or TRX1. As a result, blocking eIF4E2-GSK3β interaction promotes liver senescence that accelerates DEN-induced liver cancer under hypoxia.

Senescence is a key component of normal physiology contributing to tissue homeostasis. However, senescent cells may adversely affect tissue microenvironment through senescence-associated secretory phenotype (SASP), which promote tumor progression or age-related diseases. Hypoxia plays a critical role in the pathogenesis of diseases, potentially by inducing persistent senescence and SASP [61]. However, the relationship between hypoxia and senescence in physiological context remains unclear [11]. In this study, we found that the eIF4E2-GSK3β pathway prevented cellular senescence under physiological hypoxia by maintaining proline-directed serine/threonine phosphorylation of p53 (Fig. 7).

eIF4E2, as a homolog of eIF4E, is a well-known translational activator under hypoxia, which is essential for hypoxia adaptation [62]. Unexpectedly, our results indicated that eIF4E2-GSK3β interaction via a conserved motif regulated proline-directed kinase activity of GSK3β. Other proteins might regulate GSK3β by similar mechanism. A study showed that FRAT-GSK3β interaction regulates the proline-directed phosphorylation of FOXK, and FRAT shares the conserved GSK3β-binding motif with eIF4E2 [63, 64]. of note, a specific nanobody against the GSK3β-binding motif of eIF4E2 efficiently inhibits eIF4E2-GSK3β pathway, informing on the design of a more specific GSK3β inhibitor.

Although p53 is a canonical inducer of cellular senescence, it can also suppress senescence [65]. p53 is pivotal for establishing senescence after its activation by stress. However, whether or how physiological p53 activity regulates senescence is not quite clear [22, 65]. In current study, we found that eIF4E2-GSK3β pathway maintained the basal S/T-P phosphorylation of p53, which enabled p53 to prevent senescence under physiological condition. Multiple site S/T-P dephosphorylations confered p53 with the transcriptional repression activity such as inhibiting the expression of TOPBP1 or TRX1 (Fig. 7). Further, p53-6A expression promoted senescence. These data suggested that basal phosphorylation of p53 was required for prevention of senescence in physiological context. However, p53-6D also promoted senescence, indicating that S/T-P phosphorylation of p53 would trigger senescence in stress context. Stress-induced phosphorylation of p53 at Ser 15 and Thr18 is known to activated senescence [66]. The perturbation of Ser18 (mouse) phosphorylation resulted in p53 physiological functional defects, thus leading to senescence [67]. Thus, the relationship between p53 phosphorylation and senescence is complex, and our observations of S/T-P phosphorylation suggested that extent of p53 phosphorylation and its cellular context further determined p53 function in modulating senescence, which warrants further investigation. In addition, we found that hypoxia inhibited the eIF4E2-GSK3β pathway to suppress the S/T-P phosphorylation of p53, suggesting that hypoxia enabled p53 to function as a transrepressor, which was consistent with previous reports [68, 69]. A peculiarity of GSK3β is its high activity in unstimulated resting cells. We also suggest that the eIF4E2-GSK3β pathway remained highly active under normal cellular conditions, which was necessary for the pivotal physiological roles of p53 by maintaining its basal S/T-P phosphorylation [20].

eIF4E2 isoforms that with GSK3β-binding motif only exist in mammals. eIF4E2 is highly correlated with hypoxia adaptation of mammals to Tibetan Plateau [70, 71]. A 63-bp insertion between the exons responsible for distinguishing the different isoforms has been reported in eIF4E2 of Tibetan, which contributes to hypoxia adaptation [71]. Based on it, we speculated that 63-bp insertion might mediate alternative splicing associated with the abundance of different eIF4E2 isoforms, and that the eIF4E2-withGβ isoform might be more abundant in high-altitude Tibetans, which would facilitate adaptation of Tibetans to hypoxic surroundings. As a proof, we found that inhibiting eIF4E2-GSK3β pathway caused liver senescence, fibrosis and tumorigenesis under physiological hypoxia. eIF4E2-GSK3β pathway played a protective role in heart against hypoxia. In addition, the eIF4E2-GSK3β pathway could regulate the S/T-P phosphorylation of Tau or HIF1α, which may underpin the effect of the eIF4E2-GSK3β pathway on hypoxia adaptation. In conclusion, we suggest that senescence modulation function of eIF4E2-GSK3β pathway is critical for hypoxia adaptation of tissues (Fig. 7).

## Supplementary information

checklist

Supplementary Information

Supplementary Figure 1

Supplementary Figure 2

Supplementary Figure 3

Supplementary Figure 4

Supplementary Figure 5

Supplementary Figure 6

Supplementary Figure 7

Supplementary Figure 8

## Data Availability

All data needed to evaluate the conclusions of this study are present in the manuscript. Requests for additional information related to this paper should be directed to the corresponding author. The RNA-Seq data: Sequence Read Archive (SRA) PRJNA605921. The iTRAQ LC-MS/MS proteomics data: iProX IPX0002016000.

## References

[1] Lee P, Chandel NS, Simon MC (2020). Cellular adaptation to hypoxia through hypoxia inducible factors and beyond. Nat Rev Mol Cell Bio.

[2] Chee NT, Lohse I, Brothers SP. mRNA-to-protein translation in hypoxia. Mol Cancer*.* 2019;18:49. 10.1186/s12943-019-0968-4 PMC6441220 30925920

[3] Cho PF, Poulin F, Cho-Park YA, Cho-Park IB, Chicoine JD, Lasko P (2005). A new paradigm for translational control: inhibition via 5'-3' mRNA tethering by Bicoid and the eIF4E cognate 4EHP. Cell.

[4] Uniacke J, Perera JK, Lachance G, Francisco CB, Lee S (2014). Cancer cells exploit eIF4E2-directed synthesis of hypoxia response proteins to drive tumor progression. Cancer Res.

[5] Uniacke J, Holterman CE, Lachance G, Franovic A, Jacob MD, Fabian MR (2012). An oxygen-regulated switch in the protein synthesis machinery. Nature.

[6] Amaya Ramirez CC, Hubbe P, Mandel N, Bethune J (2018). 4EHP-independent repression of endogenous mRNAs by the RNA-binding protein GIGYF2. Nucleic Acids Res.

[7] Chan ASL, Narita M (2019). Short-term gain, long-term pain: the senescence life cycle and cancer. Genes Dev.

[8] He S, Sharpless NE (2017). Senescence in health and disease. Cell.

[9] Lee S, Schmitt CA (2019). The dynamic nature of senescence in cancer. Nat Cell Biol.

[10] Leontieva OV, Natarajan V, Demidenko ZN, Burdelya LG, Gudkov AV, Blagosklonny MV (2012). Hypoxia suppresses conversion from proliferative arrest to cellular senescence. Proc Natl Acad Sci USA.

[11] Welford SM, Giaccia AJ (2011). Hypoxia and senescence: the impact of oxygenation on tumor suppression. Mol Cancer Res: MCR.

[12] Childs BG, Durik M, Baker DJ, van Deursen JM (2015). Cellular senescence in aging and age-related disease: from mechanisms to therapy. Nat Med.

[13] Xing J, Ying Y, Mao C, Liu Y, Wang T, Zhao Q (2018). Hypoxia induces senescence of bone marrow mesenchymal stem cells via altered gut microbiota. Nat Commun.

[14] Beurel E, Grieco SF, Jope RS (2015). Glycogen synthase kinase-3 (GSK3): regulation, actions, and diseases. Pharm Ther.

[15] Roh MS, Eom TY, Zmijewska AA, De Sarno P, Roth KA, Jope RS (2005). Hypoxia activates glycogen synthase kinase-3 in mouse brain in vivo: protection by mood stabilizers and imipramine. Biol Psychiatry.

[16] Seo YH, Jung HJ, Shin HT, Kim YM, Yim H, Chung HY (2008). Enhanced glycogenesis is involved in cellular senescence via GSK3/GS modulation. Aging Cell.

[17] Liu S, Fang X, Hall H, Yu S, Smith D, Lu Z (2008). Homozygous deletion of glycogen synthase kinase 3beta bypasses senescence allowing Ras transformation of primary murine fibroblasts. Proc Natl Acad Sci USA.

[18] Kim YM, Song I, Seo YH, Yoon G (2013). Glycogen synthase kinase 3 inactivation induces cell senescence through sterol regulatory element binding protein 1-mediated lipogenesis in chang cells. Endocrinol Metab.

[19] Wang SB, Venkatraman V, Crowgey EL, Liu T, Fu Z, Holewinski R (2018). Protein S-nitrosylation controls glycogen synthase kinase 3beta function independent of its phosphorylation state. Circulation Res.

[20] Kastenhuber ER, Lowe SW (2017). Putting p53 in context. Cell.

[21] Vousden KH, Prives C (2009). Blinded by the light: the growing complexity of p53. Cell.

[22] Rufini A, Tucci P, Celardo I, Melino G (2013). Senescence and aging: the critical roles of p53. Oncogene.

[23] Kortlever RM, Higgins PJ, Bernards R (2006). Plasminogen activator inhibitor-1 is a critical downstream target of p53 in the induction of replicative senescence. Nat Cell Biol.

[24] Saito S, Yamaguchi H, Higashimoto Y, Chao C, Xu Y, Fornace AJ (2003). Phosphorylation site interdependence of human p53 post-translational modifications in response to stress. J Biol Chem.

[25] Zhang H, Shi X, Zhang QJ, Hampong M, Paddon H, Wahyuningsih D (2002). Nocodazole-induced p53-dependent c-Jun N-terminal kinase activation reduces apoptosis in human colon carcinoma HCT116 cells. J Biol Chem.

[26] Zheng H, You H, Zhou XZ, Murray SA, Uchida T, Wulf G (2002). The prolyl isomerase Pin1 is a regulator of p53 in genotoxic response. Nature.

[27] Eom TY, Jope RS (2009). GSK3 beta N-terminus binding to p53 promotes its acetylation. Mol Cancer.

[28] Zhang J, Cho SJ, Shu L, Yan W, Guerrero T, Kent M (2011). Translational repression of p53 by RNPC1, a p53 target overexpressed in lymphomas. Genes Dev.

[29] Zhang M, Zhang J, Chen X, Cho SJ, Chen X (2013). Glycogen synthase kinase 3 promotes p53 mRNA translation via phosphorylation of RNPC1. Genes Dev.

[30] Zhou H, Ye M, Dong J, Corradini E, Cristobal A, Heck AJ (2013). Robust phosphoproteome enrichment using monodisperse microsphere-based immobilized titanium (IV) ion affinity chromatography. Nat Protoc.

[31] Shilov IV, Seymour SL, Patel AA, Loboda A, Tang WH, Keating SP (2007). The Paragon Algorithm, a next generation search engine that uses sequence temperature values and feature probabilities to identify peptides from tandem mass spectra. Mol Cell Proteom: MCP.

[32] Zhang M, Zhang Y, Xu E, Mohibi S, de Anda DM, Jiang Y (2018). Rbm24, a target of p53, is necessary for proper expression of p53 and heart development. Cell Death Differ.

[33] Freemantle SJ, Portland HB, Ewings K, Dmitrovsky F, DiPetrillo K, Spinella MJ (2002). Characterization and tissue-specific expression of human GSK-3-binding proteins FRAT1 and FRAT2. Gene.

[34] Blaszczyk M, Kurcinski M, Kouza M, Wieteska L, Debinski A, Kolinski A (2016). Modeling of protein-peptide interactions using the CABS-dock web server for binding site search and flexible docking. Methods.

[35] Schwartz D, Gygi SP (2005). An iterative statistical approach to the identification of protein phosphorylation motifs from large-scale data sets. Nat Biotechnol.

[36] Wang Z, Iwasaki M, Ficara F, Lin C, Matheny C, Wong SH (2010). GSK-3 promotes conditional association of CREB and its coactivators with MEIS1 to facilitate HOX-mediated transcription and oncogenesis. Cancer Cell.

[37] Leroy A, Landrieu I, Huvent I, Legrand D, Codeville B, Wieruszeski JM (2010). Spectroscopic studies of GSK3{beta} phosphorylation of the neuronal tau protein and its interaction with the N-terminal domain of apolipoprotein E. J Biol Chem.

[38] Flugel D, Gorlach A, Michiels C, Kietzmann T (2007). Glycogen synthase kinase 3 phosphorylates hypoxia-inducible factor 1alpha and mediates its destabilization in a VHL-independent manner. Mol Cell Biol.

[39] Zhang H, Alsaleh G, Feltham J, Sun Y, Napolitano G, Riffelmacher T (2019). Polyamines control eIF5A hypusination, TFEB translation, and autophagy to reverse B cell senescence. Mol Cell.

[40] Lane CAMaDP. (1997). p53 protein stability in tumour cells is not determined by mutation but is dependent on Mdm2 binding. Oncogene.

[41] Maclaine NJ, Hupp TR (2009). The regulation of p53 by phosphorylation: a model for how distinct signals integrate into the p53 pathway. Aging (Albany NY).

[42] Buschmann T, Potapova O, Bar-Shira A, Ivanov VN, Fuchs SY, Henderson S (2001). Jun NH2-terminal kinase phosphorylation of p53 on Thr-81 is important for p53 stabilization and transcriptional activities in response to stress. Mol Cell Biol.

[43] Wei G, Liu G, Liu X (2003). Identification of two serine residues important for p53 DNA binding and protein stability. FEBS Lett.

[44] Qu L, Huang S, Baltzis D, Rivas-Estilla AM, Pluquet O, Hatzoglou M (2004). Endoplasmic reticulum stress induces p53 cytoplasmic localization and prevents p53-dependent apoptosis by a pathway involving glycogen synthase kinase-3beta. Genes Dev.

[45] Jeon Y, Ko E, Lee KY, Ko MJ, Park SY, Kang J (2011). TopBP1 deficiency causes an early embryonic lethality and induces cellular senescence in primary cells. J Biol Chem.

[46] Young JJ, Patel A, Rai P (2010). Suppression of thioredoxin-1 induces premature senescence in normal human fibroblasts. Biochemical biophysical Res Commun.

[47] Hata AN, Engelman JA, Faber AC (2015). The BCL2 family: key mediators of the apoptotic response to targeted anticancer therapeutics. Cancer Discov.

[48] Yang M, Wang L, Wang X, Wang X, Yang Z, Li J (2017). IL-6 promotes FSH-induced VEGF expression through JAK/STAT3 signaling pathway in bovine granulosa cells. Cell Physiol Biochem..

[49] Wang HC, Chen CW, Yang CL, Tsai IM, Hou YC, Chen CJ (2017). Tumor-associated macrophages promote epigenetic silencing of Gelsolin through DNA methyltransferase 1 in gastric cancer cells. Cancer Immunol Res.

[50] Khan A, Fornes O, Stigliani A, Gheorghe M, Castro-Mondragon JA, van der Lee R (2018). JASPAR 2018: update of the open-access database of transcription factor binding profiles and its web framework. Nucleic Acids Res.

[51] Tanaka T, Williams RL, Rabbitts TH (2007). Tumour prevention by a single antibody domain targeting the interaction of signal transduction proteins with RAS. Embo J.

[52] McMahon C, Baier AS, Pascolutti R, Wegrecki M, Zheng S, Ong JX (2018). Yeast surface display platform for rapid discovery of conformationally selective nanobodies. Nat Struct Mol Biol.

[53] Chung CI, Zhang Q, Shu X (2018). Dynamic imaging of small molecule induced protein–protein interactions in living cells with a fluorophore phase transition based approach. Anal Chem.

[54] Zhang Q, Huang H, Zhang L, Wu R, Chung CI, Zhang SQ (2018). Visualizing dynamics of cell signaling in vivo with a phase separation-based kinase reporter. Mol Cell.

[55] Abat JK, Saigal P, Deswal R (2008). S-Nitrosylation—another biological switch like phosphorylation?. Physiol Mol Biol Plants..

[56] Wang YX, Lim SL, Pang CC (1995). Increase by NG-nitro-L-arginine methyl ester (L-NAME) of resistance to venous return in rats. Br J Pharmacol.

[57] Konermann S, Lotfy P, Brideau NJ, Oki J, Shokhirev MN, Hsu PD (2018). Transcriptome engineering with RNA-targeting type VI-D CRISPR effectors. Cell.

[58] Yuan G, Nanduri J, Bhasker CR, Semenza GL, Prabhakar NR (2005). Ca2+/calmodulin kinase-dependent activation of hypoxia inducible factor 1 transcriptional activity in cells subjected to intermittent hypoxia. J Biol Chem.

[59] Li F, Huangyang P, Burrows M, Guo K, Riscal R, Godfrey J (2020). FBP1 loss disrupts liver metabolism and promotes tumorigenesis through a hepatic stellate cell senescence secretome. Nat Cell Biol.

[60] Shin M, Lee HA, Lee M, Shin Y, Song JJ, Kang SW (2018). Targeting protein and peptide therapeutics to the heart via tannic acid modification. Nat Biomed Eng.

[61] Yeo EJ (2019). Hypoxia and aging. Exp Mol Med.

[62] Melanson G, Timpano S, Uniacke J (2017). The eIF4E2-directed hypoxic cap-dependent translation machinery reveals novel therapeutic potential for cancer treatment. Oxid Med Cell Longev.

[63] He L, Gomes AP, Wang X, Yoon SO, Lee G, Nagiec MJ (2018). mTORC1 promotes metabolic reprogramming by the suppression of GSK3-dependent Foxk1 phosphorylation. Mol Cell.

[64] He L, Fei DL, Nagiec MJ, Mutvei AP, Lamprakis A, Kim BY (2019). Regulation of GSK3 cellular location by FRAT modulates mTORC1-dependent cell growth and sensitivity to rapamycin. Proc Natl Acad Sci USA.

[65] Demidenko ZN, Korotchkina LG, Gudkov AV, Blagosklonny MV (2010). Paradoxical suppression of cellular senescence by p53. Proc Natl Acad Sci USA.

[66] Webley K, Bond JA, Jones CJ, Blaydes JP, Craig A, Hupp T (2000). Posttranslational modifications of p53 in replicative senescence overlapping but distinct from those induced by DNA damage. Mol Cell Biol.

[67] Armata HL, Garlick DS, Sluss HK (2007). The ataxia telangiectasia-mutated target site Ser18 is required for p53-mediated tumor suppression. Cancer Res.

[68] Feng X, Liu X, Zhang W, Xiao W (2011). p53 directly suppresses BNIP3 expression to protect against hypoxia-induced cell death. EMBO J.

[69] Koumenis C, Alarcon R, Hammond E, Sutphin P, Hoffman W, Murphy M (2001). Regulation of p53 by hypoxia: dissociation of transcriptional repression and apoptosis from p53-dependent transactivation. Mol Cell Biol.

[70] Zhang B, Ban DM, Gou X, Zhang YW, Yang L, Chamba Y, et al. Genome-wide DNA methylation profiles in Tibetan and Yorkshire pigs under high-altitude hypoxia. J Anim Sci Biotechnol. 2019;10:25. 10.1186/s40104-019-0316-y PMC6397503 30867905

[71] Ouzhuluobu HY, Lou H, Cui C, Deng L, Gao Y (2020). De novo assembly of a Tibetan genome and identification of novel structural variants associated with high-altitude adaptation. Natl Sci Rev.

