# Supplementary material for: Mammalian eIF4E2-GSK3β maintains basal phosphorylation of p53 to resist senescence under hypoxia
Source: Cell Death Dis. 2022 May 14;13(5):459. doi: 10.1038/s41419-022-04897-4 (PMC9107480; doi:10.1038/s41419-022-04897-4)
Supplement: Supplementary file 2 — Supplementary Information [file 41419_2022_4897_MOESM2_ESM.docx]

**Supplementary Information For**

Mammalian eIF4E2-GSK3β maintains basal phosphorylation of p53 to resist

senescence under hypoxia

**Materials and methods**

**Reagent or resource**

The phospho-specific antibodies p-RBM38#1 and p-RBM38#2 was generated by immunizing rabbits with the synthetic phospho-peptide span serine195 [^187^YDQYPYAAS(p)PAT^198^] or [^195^S(p)PATAASFVGYS^206^]. The phospho-specific

p-p53(S127) antibodies were generated by immunizing rabbits with the synthetic phospho-peptide span serine 127 [^124^CTYS(p)PALNK^132^]. The RBM38 antibodies were generated by immunizing rabbits with the peptide [^195^SPATAASFVGYS^206^]. The phospho-specific p-HIF1α(S589) antibodies were generated by immunizing rabbits with the synthetic phospho-peptide span serine 589 [^584^ESSSAS(p)PES^592^]. The antibodies recognize the GSK3β binding motif of eIF4E2 were generated by immunizing rabbits with the peptide [^231^RLLFQNLWKPRL^242^].

| REAGENT | SOURCE | IDENTIFIER |
| --- | --- | --- |
| Antibodies | | |
| β-tubulin | Sigma Aldrich | T5201 |
| Actin | Sigma Aldrich | F3022 |
| GAPDH | ABclonal | AC001 |
| p53 (1C12) | Cell Signaling Technology | #2524 |
| p53 | GeneTex | GTX128135 |
| p53 | Santa Cruz Biotechnology | sc-393031 |
| p53 | ABclonal | A5761 |
| p53(zebrafish) | abcam | ab77813 |
| p-p53(Ser33) | ABclonal | AP0762 |
| p-p53(Ser46) | ABclonal | AP0476 |
| p-p53(Ser376) | abcam | ab183547 |
| p-p53(Ser315) | Cell Signaling Technology | #2528 |
| p-p53(Thr81) | Thermo Fisher | PA5-17545 |
| eIF4E2 | GeneTex | GTX82524 |
| TOPBP1 | abcam | ab2402 |
| TOPBP1 | ABclonal | A5781 |
| CDKN1A | ABclonal | A1483 |
| TRX1  FRAT1 | ABclonal  ABclonal | A7638  A16093 |
| MDM2 | Abcam | ab16895 |
| BCL2 | ABclonal | A19693 |
| BAD | ABclonal | A19595 |
| FLAG-Tag | Sigma Aldrich | F4799 |
| HA-Tag  GST-Tag | ABclonal  ABclonal | AE008  AE001 |
| HIS-Tag | ABclonal | AE003 |
| Histone | ABclonal | A4835 |
| p-Tau (Ser396) | ABclonal | AP0163 |
| GSK3β | Santa Cruz Biotechnology | sc-81462 |
| p-Creb1(Ser129) | ABclonal | AP0903 |
| HIF-1 alpha | Hangzhou HuaAn Biotechnology | R1510-5 |
| Mouse Control IgG | ABclonal | AC011 |
| Rabbit Control IgG | ABclonal | AC005 |
| HRP Goat Anti-Rabbit IgG | ABclonal | AS014 |
| HRP Goat Anti-Mouse IgG | ABclonal | AS003 |
| Kit | | |
| Senescence β-Galactosidase Staining Kit | Beyotime（China） | C0602 |
| Mouse Interleukin-1β ELISA Kit | Beyotime（China） | PI301 |
| Mouse Interleukin-6 ELISA Kit | Beyotime（China） | PI326 |
| Mouse Interleukin-8 ELISA Kit | MYBiosource | MBS7606860 |
| Click-iT Plus OPP Protein Synthesis Assay | Thermo Fisher | C10456 |
| Click-iT^®^Metabolic Labeling kits | Thermo Fisher | C10102，C10276 |
| mMessage mMachine SP6 kit | Thermo Fisher | AM1340 |
| Chemicals | | |
| Cycloheximide | MedChemExpress | HY-12320 |
| Nocodazole | MedChemExpress | HY-13520 |
| Camptothecin | MedChemExpress | HY-16560 |

**Peptide sequence** Designed online ([www.mimotopes.com](http://www.mimotopes.com)), Peptide e2-I: YGRKKRRQRRRRLLFQNLWKPRL, consists of two parts, the core is the GSK3β binding motif of eIF4E2: RLLFQNLWKPRL, which then fused with penetrating peptide for cell treatment [[1](#_ENREF_1)]. And the corresponding scramble peptide e2-S: YGRKKRRQRRRFLWRLRKNQLPL. Another e2-I/e2-S is the core sequence fused with different penetrating peptide RQIKIWFQNRRMKWKK for intraperitoneal injection of mice [[2](#_ENREF_2)]. The core sequence of peptide G3-I is the eIF4E2 binding motif of GSK3β: CSRLLEYTPTARL, fused with above penetrating peptide. And the core sequence of corresponding scramble peptide G3-S is RLLYTPSCATLRE.

**Nanobody sequence**

Nb-28A1: ......SGRTFSSY (CDR1) ......EFVAAISWGSGITNYA (CDR2) ......NYLPDLYD (CDR3) ......

**Plasmids** To generate PGEX6p-1 vector expressing GST-tagged RBM38, eIF4E2 (human isoform A), GSK3β, FRAT1, a DNA fragment was amplified using cDNA samples from HCT116 cells as a template with a pair of specific primers. The PCR product was inserted into PGEX6p-1 vector through E*co*RI and XhoI sites and confirmed by sequencing. The specific primers for GST-tagged RBM38 were a forward primer, 5' GAC TGA ATT CAT GCT GCT GCA GCC CGC TCC 3′, and a reverse primer, 5′ GAC TCT CGA GTT ACT GCA TCC GGT CAG GCT 3′. The specific primers for eIF4E2 were a forward primer, 5′ GAC TGA ATT CAT GAA CAA CAA GTT CGA CGC 3′, and a reverse primer, 5′ GAC TCT CGA GTC ATG GCA CAT TCA ACC G 3′. The specific primers for GSK3β were a forward primer, 5′ GAC TGA ATT CAT GTC AGG GCG GCC CAG AA 3′, and a reverse primer,

5′ GAC TCT CGA GTC ACG TCG AGT TGG AAG CTG ATG C 3′. The specific primers for FRAT1 were a forward primer, 5′ GAC TGA ATT CAT GCC GTG CCG GAG GGA GGA 3′, and a reverse primer, 5′ GAC TCT CGA GTT AGC TGC CAG GCA CAA GAA 3′.

To generate pET28a vector expressing His-tagged RBM38, eIF4E2, GSK3β, a DNA fragment was amplified using cDNA samples from HCT116 cells as a template with a pair of specific primers. To generate pET28a vector expressing His-tagged Nb-28A1, Nb-BV025. A DNA fragment was amplified using gene synthesis (genecreate) as a template. The PCR product was inserted into pET28a vector through E*co*RI and XhoI sites and confirmed by sequencing. His-tagged RBM38 eIF4E2 and GSK3β specific primers are consistent with those described above. The specific primers for His-tagged Nb-28A1 were a forward primer, 5′ GAC TGA ATT CAT GCA GGT GCA GCT GCA GGA GTC 3′, and a reverse primer, 5′GAC TCT CGA GCT ATG AGG AGA CGG TGA CCT GAG3′. The specific primers for His-tagged Nb-BV025 were a forward primer, 5′ GAC TGA ATT CAT GCA AGT TCA ACT TCA AGA ATC 3′, and a reverse primer, 5′GAC TCT CGA GCT ACG AAG AAA CAG TAA CTT GAG 3′. To generate pET28a vector expressing His-tagged HIF1α and HIF1α(S589A) a DNA fragment was amplified using cDNA samples from HCT116 cells as a template with a pair of specific primers. The PCR product was inserted into pET28a vector through BamHI and SacI sites and confirmed by sequencing. The specific primers for His-tagged HIF1α were a forward primer, 5' GAC TGG ATC CAT GGA GGG CGC CGG CGG CGC 3′, and a reverse primer, 5′ GAC TGA GCT CTC AGT TAA CTT GAT CCA AAG 3′. The first specific primers for HIF1α(S589A) is consistent with HIF1α. The second specific primers for HIF1α(S589A) were a forward primer 5' AGT TCC GCA AGC CCT GAA AGC 3', and a reverse primer 5' GCT TTC AGG GGC TGC GGA ACT 3'.

To generate PGEX6p-1 vector expressing GST-tagged eIF4E2, deletion mutants (Δ231-243), and GSK3β (Δ314-329), PCR products were amplified using overlapping extension method and GST-tagged eIF4E2 or GSK3β as templates. The specific primers for eIF4E2 were a forward primer 5' GAC TGG ATC CAT GAA CAA CAA GTT CGA CGC 3 ', and a reverse primer 5' GAC TCT CGA GTC ATG GCA CAT TTT GGG GGC CCA GCC TGC CT 3'. These PCR products were inserted into the pGEX6p-1 vector through the BamHI and XhoI sites and confirmed by sequencing. The first specific primers for GSK3β were a forward primer 5' GAC TGA ATT CAT GTC AGG GCG GCC CAG AA 3' and a reverse primer 5' CCA GTG GTG TTG CCT CCG GTG GAG T3'. The second specific primers for GSK3β were a forward primer 5' ACC GGA GGC AAC ACC ACT GGA AGC T 3 ', and a reverse primer 5' GAC TCT CGA GTC ACG TCG AGT TGG AAG CTG ATG C 3'. These PCR products were inserted into the pGEX6p-1 vector through the E*co*RI and XhoI sites and confirmed by sequencing.

To generate vector PGL3-TOPBP1 (5'UTR, -5841, -4865) or PGL3-TOPBP1(ΔRRs) (Δp53 binding site), a template gene was synthesized (genecreate), and a DNA fragment was inserted into the pGL3-Basic vector through the KpnI and XhoI sites, and confirmed by sequencing. To generate vector PGL3-TRX1, a template gene was synthesized (genecreate), and a DNA fragment was inserted into the

pGL3-Basic vector through the BmtI and HindIII sites, and confirmed by sequencing.

To generate (FLAG or EGFP-tagged) p53-WT or p53-6A mutants (S33A, S46A, T81A, S127A, T150A, S315A) or p53-6D mutants (S33D, S46D, T81D, S127D, T150D, S315D) and FLAG-GSK3β. a DNA fragment was amplified using gene synthesis (genecreate) as a template with a forward primer, 5' GAC TGG ATC CAT GGA GGA GCC GCA GTC AGA 3', and a reverse primer, 5′GAC TCT CGA GTC AGT CTG AGT CAG GCC CTT CT 3′. The DNA fragment was inserted into pcDNA3-3xFLAG vector or pcDNA3-EGFP vector through BamHI and XhoI sites and confirmed by sequencing. GSK3β specific primers are consistent with those described above. The pcr products were inserted into pcDNA3-3xFLAG vector through BamHI and XhoI sites and confirmed by sequencing.

To generate HA-tagged Nb-28A1or Nb-BV025. A DNA fragment was amplified using gene synthesis (genecreate) as a template. The specific primers for Nb-28A1 were a forward primer, 5' GAC TGG TAC CAT G CA GGT GCA GCT GCA GGA GTC 3', and a reverse primer, 5′ GAC TGG ATC CCT ATG AGG AGA CGG TGA CCT GAG 3′. The specific primers for Nb-BV025 were a forward primer, 5' GAC TGG TAC CAT GCA AGT TCA ACT TCA AGA ATC 3' and a reverse primer,

5′ GAC TGG ATC CCT ACG AAG AAA CAG TAA CTT GAG 3′. To generate HA-tagged eIF4E2. a DNA fragment was amplified using GST-eIF4E2 as a template with a forward primer, 5′ GAC TGG TAC CAT GAA CAA CAA GTT CGA CGC 3′, and a reverse primer, 5′ GAC TGG ATC CTC ATG GCA CAT TCA ACC G 3′. The DNA fragment was inserted into pcDNA3-HA vector through KpnI and BamHI sites and confirmed by sequencing.

To generate pcDNA3-zebrafish p53-4A mutants (T65A, S96A, T151A, S355A). a DNA fragment was amplified using gene synthesis (genecreate) as a template with a forward primer, 5' GAC TGC TAG CAT GGC GCA AAA CGA CAG CCA 3', and a reverse primer, 5′GAC TGG ATC CTT AAT CAG AGT CGC TTC TTC 3′. The DNA fragment was inserted into pcDNA3 vector through NheI and BamHI sites and confirmed by sequencing.

To generate pAAV-TOPBP1, a DNA fragment was amplified using gene synthesis (genecreate) as a template with a forward primer, 5' GAC TCC GCG GAT GTC CAG AAA TGA CAA AGA 3', and a reverse primer, 5′ GAC TGG ATC CTT AGT GTA CTC TAG GTC GTT 3′. To generate pAAV-TRX1 a DNA fragment was amplified using gene synthesis (genecreate) as a template with a forward primer, 5' GAC TCC GCG GAT GGT GAA GCA GAT CGA GAG C 3', and a reverse primer, 5′ GAC TGG ATC CCT AGA CTA ATT CAT TAA TGG TGG CT 3′. To generate pAAV-Nb-28A1 a DNA fragment was amplified using pcDNA3-HA-Nb-28A1 as a template with a forward primer, 5' GAC TCC GCG GAT GTA CCC ATA CGA TGT TCC AGA 3', and a reverse primer, 5′ GAC TGG ATC CCT ATG AGG AGA CGG TGA CCT GAG 3′. To generate pAAV-Nb-BV025 a DNA fragment was amplified using pcDNA3-HA-Nb-BV025 as a template with a forward primer, 5' GAC TCC GCG GAT GTA CCC ATA CGA TGT TCC AGA 3', and a reverse primer, 5′ GAC TGG ATC CCT ACG AAG AAA CAG TAA CTT GAG 3′. The DNA fragment was inserted into pAAV-CMV-eGFP vector through SacII and BamHI sites and confirmed by sequencing.

To generate pminiTol2-5UAS-eIF4E2-Sg1180, a DNA fragment was amplified using plasmid GST-tagged eIF4E2 as a template with a forward primer, 5' GAC TGA ATT CAT GAA CAA CAA GTT CGA CGC 3', and a reverse primer, 5′ GAC TTC TAG ATC ATG GCA CAT TCA ACC GCG 3′. The DNA fragment was inserted into pminiTol2-5UAS- tdTOM-Sg1180 vector through E*co*RI and XbaI sites and confirmed by sequencing.

To generate SPPIER plasmid HOTag-eIF4E2 and GSK3β pcDNA3 vector. First, a DNA fragment was amplified using cDNA samples from HCT116 cells as a template with a forward primer of eIF4E2 5' GAC TGG ATC CAT GAA CAA CAA GTT CGA CGC 3', and a reverse primer 5' GAC TGA ATT CTG GCA CAT TCA ACC GCG 3'. PCR products were inserted into pcDNA3-Tag3 vector through BamHI and EcoRI sites. Second a DNA fragment was amplified using plasmid DNA from GST-tagged-GSK3β as a template with a pair of specific primers. The specific primers for GSK3β were a forward primer, 5' GAC TGG TAC CAT GTC AGG GCG GCC CAG AA 3', and a reverse primer 5' GAC TAC CGG TGG TGG AGT TGG AAG CTG ATG CAG 3'. PCR products were inserted into the constructed pcDNA3-eIF4E2-Tag3-Tag6 through KpnI and AgeI sites, and confirmed by sequencing.

**Western blotting analysis and co-immunoprecipitation (Co-IP)** Western blotting analysis was performed as previously described [[3](#_ENREF_3)]. Cell lysates suspended in 2xSDS loading buffer were resolved by SDS-PAGE, transferred to a PVDF membranes, and probed with indicated antibodies. The immunoreactive bands were visualized by the enhanced chemiluminescence (Pierce) and quantified by densitometry with ChemiScope 6000 Exp (Chemi, CHN). Immunoprecipitation assay was performed. Briefly, cells were lysed in 0.5 % Triton lysis buffer (25 mM Tris pH 7.5, 25 mM NaCl, 1 % Triton X-100) supplemented with the proteinase inhibitor cocktail (100μg/ml), followed by incubation with 1 μg of antibody or control IgG. The immunocomplexes were brought down by protein A/G beads and subjected to Western blotting analysis.

**GST pull-down assay** GST pull-down assay was performed as previously described [[3](#_ENREF_3)]. The recombinant His or GST tagged proteins were expressed in bacteria BL21 and purified by using Ni-NTA(Qiagen) and glutathione sepharose beads (GenScript), respectively. For GST pull-down assay, 1nmol of recombinant His-tagged proteins and 1nmol of recombinant GST-tagged proteins were incubated in GST pull-down Buffer (50 mM HEPES pH 7.5, 50 mM NaCl, 2 mM EDTA PH 8.0, 0.1% Nonidet P-40, and 10% glycerol) for 2 hours at 4°C, followed by precipitation with glutathione-sepharose beads for 30 min at 4°C. After three washes, beads were resuspended in 2xSDS loading buffer and subjected to Western blotting analysis.

**In vitro kinase assay** In vitro kinase assay was performed as previously described [[3](#_ENREF_3)]. His-tagged HIF1α, HIF1α (S589A) and eIF4E2 were expressed in bacteria BL21 and purified with Ni-NTA beads (30210, Qiagen). 100ng of each purified protein was incubated in the presence or absence of 500ng M2 FLAG antibody purification Flag-GSK3β in a reaction buffer (50 mM Tris–HCl, pH 7.5, 50 mM MgCl_2_, 1 mM DTT, 500 μM ATP, 5% glycerin) at 30 °C for 45 min. The reaction mixtures were subjected to SDS-PAGE, and Western blotting analysis.

**RNA isolation and reverse transcription-PCR** Total RNA was extracted using TRIzol (Invitrogen) according to manufacturer’s instructions. M-MuLV reverse transcription (Takara) was used for mRNA measurements. In brief, RT was performed using the ExScript RT reagent kit (Takara) in a final volume of 20 μl containing 1 μg total RNA, 4 μl 5X ExScript buffer, 1 μl deoxynucleotide triphosphate (dNTP, 10 μM) mixture, 1 μl oligo(dT) primer, 0.5 μl ExScript RTase, 0.5 μl RNase inhibitor and RNase-free water. PCR was conducted according to the instructions of TsingKe Golden mix under the following conditions: pre-DNA denaturation at 98ºC for 2 min; DNA denaturation at 98ºC for 10 sec; annealing for 30 sec at 60ºC; elongation was carried out at 72ºC for 10 sec; the total cycle number was 30. All experiments were performed in triplicate. The primers were as follows: human TOPBP1 (forward: 5′ TGC TAC CTG GTC ATT CTG TAC C 3′, reverse: 5′ AGT GTA CTC TAG GTC GTT TGA TT 3′), human TRX1 (forward: 5′ TGC TTT TCA GGA AGC CTT GGA 3′, reverse: 5′ GAC TAA TTC ATT AAT GGT GGC TTC A 3′), human GSN (forward: 5′ AAC CGC AAG CTG GCC AAG CT 3′, reverse:

5′ TCC ATC TTG GTG ATG AAG TCA G 3′), human VEGF (forward: 5′ TGT GAA TGC AGA CCA AAG 3′, reverse: 5′ GCC TTG CAA CGC GAG TCT GTG 3′), human HNRNPD (forward: 5′ AAG AAA TAC CAC AAT GTT GG 3′, reverse:

5′ CCA TAT CCA TAG TAG TTG TTG 3′), human EEF2 (forward: 5′ CGT CAT CAT CTC CAC CTA CG 3′, reverse: 5′ CTT CTT CAT CAT GTC CTC TAC 3′), human GAMT (forward: 5′ CAC GGC AGA CAC ACA AGG TCA T 3′, reverse:

5′ GGT GAT GTC TGA GTA CTT GGA C 3′), human NEK9 (forward: 5′ CCG TCA GGT GTC ATG TGG TG 3′, reverse: 5′ TGG ACA GAG ACA AGG CTA 3′), human BCL2 (forward: 5′ AGA GCG TCA ACC GGG AGA TGT CG 3′, reverse:

5′ TCA CTT GTG GCC CAG ATA GGC A 3′), BAD (forward: 5′ AAC CAG CAG CAG CCA TCA TGG 3′, reverse: 5′ GGC GAG GAA GTC CCT TCT T 3′), human β-ACTIN (forward: 5′ AGA CCT GTA CGC CAA CAC AG 3′, reverse: 5′ CGG ACT CGT CAT ACT CCT GC 3′), zebrafish TOPBP1 (forward: 5′ CCT AAT GTT ATT GTT GTG CAG GGA 3′, reverse: 5′ AGC TGA AGC CTC ACT GAT GTC 3′), zebrafish TRX1 (forward: 5′ CTC ATG CAT GCC AAC ATT CC 3′, reverse: 5′ ACA AAA CAT ATA AAT GTA GCT GTC A 3′), zebrafish β-ACTIN (forward: 5′ CCA CCA TGT ACC CTG GCA TT 3′, reverse: 5′ CCA CCA TGT ACC CTG GCA TT 3′). β-ACTIN mRNA was used as an internal control. Real-time PCR was performed in a CFX96 Touch (BIO-RAD) according to the manufacturer’s instructions. Normalization was made to total RNA and to-actin mRNA with similar results. The data presented are the ones normalized to-actin transcripts. The results are statistically analyzed using graphpad 8.0. primers are consistent with those described above.

**Chromatin Immunoprecipitation (ChIP) Assay** FLAG tagged p53 were overexpressed in p53-null HCT116 cells for 36 hours. The cells were washed with chilled PBS, then incubated in 1% formaldehyde for 15 min at room temperature for chromatin cross-linking. The cross-linking reaction was then terminated by adding 141 μL of 1 M glycine per 1 mL of culture medium to a final concentration of 125 mM and incubated at room temperature for 10 minutes. Place the plate on the top of ice and wash 3 times with 10 mL ice-cold PBS**/**0.5 mM PMSF. Scrape cells in 10 mL ice-cold PBS**/**0.5 mM PMSF. Centrifuge at 1000 rpm for 5 minutes in cold centrifuge. Discard the supernatant, add 1 mL cold cell lysis buffer (10 mM EDTA pH 8.0, 50 mM Tris-HCl pH 8.0, 1% SDS, and protease inhibitors) per 1**×**10^7^ cells. Resuspend the pellet by pipetting up and down for several times, and incubate on ice for 10 minutes. Sonicated in Diagenode water bath-sonicator for 6 minutes to shear the chromatin into 150bp fragment. Avoid foam and keep the sample on ice, and then centrifuged at 14000 rpm at 4°C for 10 min, and retain the supernatant. 50-150 ug of ultrasound chromatin were diluted 10-fold in ChIP dilution buffer (1%SDS,1% Triton-X-100, 2 mM EDTA pH 8.0, 16.7 mM Tris-HCl pH 8.0, and 167 mM NaCl), and incubated with 50 ul anti-FLAG antibody resin (F1804, Sigma) at 4°C for 4 hours with rotation. Wash the beads for one times with low-salt buffer (0.1% SDS, 1% Triton X-100, 2 mM EDTA pH 8.0, 20 mM Tris-HCl pH 8.0, and 150 mM NaCl), twice in high-salt solution buffer (0.1% SDS, 1% Triton-X-100, 2 mM EDTA pH 8.0, 20 mM Tris-HCl pH 8.0, and 500 mM NaCl), twice in LiCl buffer (0.25 M LiCl, 1% Nonidet P-40, 1% sodium deoxycholate, 1 mM EDTA pH 8.0 , 10 mM Tris-HCl pH 8.0), and twice in TE buffer (1M Tris-HCl pH 8.0), discard the supernatant. Incubate the beads from the previous step with 100 μL of elution buffer for 15 minutes at room temperature, repeat the elution once, and finally 200 ul of elution buffer (1% SDS, 0.1 M NaHCO3). Add a final concentration of 0.2M NaCl, incubate at 65 °C for overnight, to reverse cross-link chromatin. The DNA was then submitted to RNase and proteinase K digestion and purify DNA using a DNA purification kit (DP204, TIANGEN). A total of 1 % of each IP was used as a template to perform PCR using primers specific for a region of interest. Primers for TOPBP1 RE1 region are forward primer 5' CGA TCT TCT GAC CTC ATG ATC TAC C 3' and a reverse primer

5' AAG CAA AAG ACA AGC TCA GGG 3'. Primers for TOPBP1 RE2 region are forward primer 5' TGG GTG AAT CAC AAG ATC AGG AG 3' and a reverse primer 5' TTC AAG CGA TTC TTC TGC CTC A 3'. Primers for TOPBP1 region without RE are forward primer 5' CAT GTT GGT CAG GCT GGT CTC 3' and a reverse primer 5' GTG ACA TTA TGG GCA ACT TTA TCT G 3'. Primers for TRX1 RE region are forward primer 5' CTC CAC AAG GCC AGC TAA CA 3' and a reverse primer 5' ACA CTT GTG TGA GAT TCT TCT AAC A 3'. Primers for TRX1 region without RE are forward primer 5' CAT ACA GCT CCT TCC TCT CTG C 3' and a reverse primer 5' GAT ATA GGC AAG GGA CAC ATT C 3'. Primers for GAPDH of negative control are forward primer 5' CTA CTA GCG GTT TTA CGG GCG C 3' and a reverse primer 5' GGA GGC TGC GGG CTC AAT TT 3', Primers for P21 of positive control are forward primer 5' CCC CAC AGC AGA GGA GAA AG 3' and a reverse primer 5' GAA CCC AGG CTT GGA GCA G 3'.

**Dual luciferase reporter assays** The TOPBP1 promoter region was cloned into pGL3-Basic luciferase reporter plasmid. p53-null HCT116 cells were transfected with 500ng of luciferase reporter plasmid (pGL3-TOPBP1 or pGL3-Basic) and 500ng of vectors expressing p53 and 25 ng of internal control pRL-CMV. Cells were grown in 24-well plate (Corning). After 48 hours, cells were collected and luciferase activity was evaluated by the Dual-Luciferase Reporter Assay System [[4](#_ENREF_4)]. Cells were lysed for 15 minutes at room temperature by mixing with 100 μL of the passive lysis buffer (PLB) provided in the Dual Luciferase Reporter Assay System kit (E1910, Promega), and the cell debris was then pelleted by centrifugation at 7500**×**g for 1 minute. 20 μL of the supernatant was then loaded into the wells of a white flat-bottomed Costar

96-well plate (Corning). 100 μL of Dual-Glo Luciferase assay reagent was added to each well, followed by the addition of 100 μL Dual-Glo Stop & Glo reagent. Assays were performed using a Cytaion5 microplate reader (Bio-Tech, USA). Renilla luminescence was normalized to the internal control vector pRL-CMV luminescence. Promoter activities were measured as relative luminescence units (RLU) where the value of the firefly luciferase luminescence of pGL3-Basic was divided by the Renilla luciferase pRL-CMV from the same well. GraphPad Prism 8 software was used for statistical analysis.

**Senescence-associated β-galactosidase (SA-β-Gal) assay** Cells were plated in 12-well plates and treated with 5 μM e2-I or e2-S for 96 hours. Then, senescence-associated β-galactosidase (SA-β-Gal) staining was performed according to the manufacturer’s instructions (senescence β-galactosidase staining kit, Beyotime, China). After removed the cell culture solution, rinsed the plate one time with 500μL 1×PBS (HyClone), add 500μl of β-galactosidase staining fixative, and fix for 15 minutes at room temperature. Rinsed the plate three times with 500μL 1×PBS 3 minutes each. Add 600μL of the β-Galactosidase Staining Solution (β-Galactosidase Staining A 6μL, B 6μL, C 558μL, X-Gal Solution 30μL). Incubated the plate at 37°C at least overnight in a dry incubator (no CO_2_). Removed the staining working solution, and 500μl of PBS can be added. Observe under inverted microscope (DMi8 Lecia).

**The SA-β-Gal staining for tissues** tissues block or Cryosections were fixed with 4% Paraformaldehyde in PBS for 30 min (Cryosections) or 12h (tissues block). Washed with 1×PBS and stained for 8-12 h in 1mL of the β-Galactosidase Staining Solution (β-Galactosidase Staining A 10μL, B 10μL, C 930μL, X-Gal Solution 50μL). The slides were then rinsed in 1×PBS, counterstained with 0.1% Nuclear fast red (Sigma) for 5 min and mounted for examination.

**RNA-Seq experiment** HCT116 and p53-null HCT116 cells were treated with 5μM e2-I or e2-S for 24 hours. Each sample group has three biological replicates. A small aliquot of each sample was subjected to Western blotting to confirm the inhibitory effect of e2-I on the phosphorylation of RBM38 S195. Stranded paired-end RNA sequencing (RNA-seq) with 100 bp read lengths was performed using a HiSeq2500 (Illumina) according to the manufacturer’s protocol. The acquired sequence reads were aligned to the human genome sequence (hg38) by HISAT2 (version 2.1.0) [[5](#_ENREF_5)]. The number of reads per gene was determined using featureCounts v1.6.2 (Scr vs e2-I samples) [[6](#_ENREF_6)]. Differentially expressed genes were determined using DEseq [[7](#_ENREF_7)]. GSEA was performed using the pre-ranked module within the GSEA 4.03 software (Broad Institute) [[8](#_ENREF_8)]. Genes were ranked by differential expression between scramble peptide and e2-I treated samples and run against C2 KEGG gene sets. Identification of differential expression of senescence-related genes, by referring to database of senescence-genes including CSGene or Cellage (genomics.senescence.info/cells/).

**AHA Labeling of Nascent Proteins** AHA Labeling assay was performed as previously described [[9](#_ENREF_9)]. AHA Labeling of Nascent Proteins Click-iT Metabolic Labeling kits (C10102, C10276, Thermo Fisher) were used for AHA labeling according to manufacturer’s protocol. Briefly, cells were pre-cultured in methionine-free DMEM for 1 hour, then 50 μM AHA was added for 1 hour. Cells were lysed in lysis buffer (50 mM Tris-HCl, pH 8.0, 1% SDS with protease**/**phosphatase inhibitors). The cleared cell lysate (200 mg protein**/**sample) was used to perform Click reaction for biotin labeling. Biotinylated proteins were pulled down by streptavidin-agarose beads (50 μL beads/sample, S1638, Sigma) overnight to purify nascent proteins for Western blotting analysis.

**The construction of a synthetic yeast display nanobody library** Nanobody

library construction was performed as previously described [[10](#_ENREF_10)].  Briefly, the DNA library of nanobodies was constructed by two-step overlap-extension PCR (OE-PCR). A set of ten primers (Tsingke Biotechnology Co., Ltd.) were dissolved and mixed to prepare three mixed pools, ‘mix short’, ‘mix medium’, and ‘mix long’, differed in CDR3 region of variable length of 7, 11, or 15 randomized residues respectively. The full-length nanobody DNA product from each pool was mixed in a 1:2:1 molar ratio of short/medium/long CDR3 regions, referred to as the nanobody DNA library pool hereafter, recapitulating the length distribution frequencies observed in camelid VHH domains.

The nanobody DNA library pool was successively amplified for yeast transformations with pYDSFor1-pYDSRev1, pYDSFor2-pYDSRev2, and pYDSFor3-pYDSRev3 primers. 500 mL BJ5465 yeast were grown to OD600 1.5 and transformed with 245 µg nanobody insert DNA, which had been amplified to include flanking sequences homologous to pYDS649 for recombination, and 50 µg of linearized pYDS649 plasmid. Dilutions of transformed yeast were then plated on dropout medium without tryptophan (-Trp) as single colonies to obtain an estimate of library diversity. Three cultures of 5×10^5^ yeast was inoculated in dropout medium with tryptophan and grown overnight at 30 °C for whole-library next-generation sequencing reactions. 2.5×10^9^ induced yeast for the transformation were washed and resuspended in buffer (20 mM HEPES, pH 7.5, 150 mM sodium chloride, 0.1% (w/v) ovalbumin, 1 mM EDTA) and then incubated with anti-Alexa Fluor 647 or anti-FITC microbeads (Miltenyi) at 4 °C for 30 min. Each round of MACS selection began with a preclear step which involved passing the yeast through an LD column (Miltenyi) to remove yeast-expressing nanobodies that bound nonspecifically to magnetic beads. After preclearing, eIF4E2 (231-242)-binding nanobodies were enriched over four rounds of MACS selection by staining the yeast alternately with Alexa Fluor 647-or FITC-labeled eIF4E2 (231-242) and microbeads, then passing them through an LS column (Miltenyi). During these four selection rounds, yeast was stained with successively lower concentrations of eIF4E2 (231-242): 1 μM, 250 nM, 75 nM, and 15 nM, in order to enrich for binders with higher affinities. After MACS selection, yeast was plated as single colonies, which were picked and grown as clonal populations in a 96-well plate. Following galactose induction of nanobodies, yeast was stained with Alexa Fluor 488-labeled eIF4E2 (231-242) and analyzed by flow cytometry with an Accuri C6 (BD Biosciences) to screen for nanobody binders. To achieve such specificity, yeast with reactivity to beads alone were depleted from the library before each selection step. To decrease the probability of enriching fluorophore binders, the fluorophore tag used was alternated between Alexa Fluor 647 and fluorescein isothiocyanate (FITC) for each consecutive round of magnetic selection. After four rounds of selection, single yeast colonies were isolated and stained with fluorescently labeled eIF4E2(231-242) for validation by analytical flow cytometry.

**Xenograft** Animal Experimental Ethics Committee of Huazhong Agricultural University has specifically approved the entire study, the approval # is HZAUMO-2018-014. All the mice were housed in the animal facility at Huazhong Agricultural University, and operated according to an approved protocol. The maximum allowable tumor volume is 2000 mm^3^. Peptide-treated HCT116, p53-null HCT116 cells (5**×**10^6^ cells) mix Membrane Matrix (A1413301, Gibco) in a 1: 1 ratio was suspended and injected subcutaneously to the left and right sides of the thigh (6-week-old, BALB/C Nude mice, female). After tumor formation, tumor mass is measured at 21 days.

**Production of adeno-associated viruses** Adeno-associated viruses were generated using packaging plasmids pAAV-Rep2/Cap8 and pAAV-helper

(Cell Biolabs, Inc) together with pAAV-CMV-eGFP. Briefly, AAV8-TOPBP1 or (AAV8-TRX1, AAV8-Nb-28A1, AAV8-Nb-BV025) were co-transfected with pAAV-Rep2/Cap8 and pAAV-helper into 293T cells, respectively, by a calcium phosphate-based protocol. Viral particles were harvested following 48h of transfection. After purification by CsCl gradient centrifugation, the titers of viral particles were determined by quantitative real-time PCR and these vectors were applied in the following animal experiments. AAV8-TOPBP1 or others (1×10^11^viral genomes in 100μl saline) were intravenous injected into mice. 2 weeks later, mice were treated intraperitoneally with 35mg/kg peptide e2-I or scrambled e2-S every two days.

**Open field test** After consecutive intraperitoneal injection of 35mg/kg e2-I or scrambled e2-S every day for 2 days, the mice were housed at normoxia or physiological hypoxia (12% O_2_) conditions. on the 3^th^ or 6^th^ day, the mice were subjected to an open field test. The activity was measured as the total distance traveled (meters) and rearing times in 5 min in the open field chamber

(40 cm long×40 cm wide×25 cm high). The center square of the open field, comprising 25% of the total area, was defined as the “central area” of the open field. Mice used for the test were 8 weeks old.

**SPPIER assay** SPPIER (separation of phases-based protein interaction reporter) assay is based on multivalent PPI-induced protein phase transition, which leads to formation of highly concentrated protein droplets [[11](#_ENREF_11), [12](#_ENREF_12)]. Cells were grown in 35 mm glass bottom microwell (20 mm) dishes (801001, NEST). The constructed SPPIER plasmid is transfected into HCT116 cells for 24 hours, followed by hypoxia (1% O_2_) or L- Arginine treatment. Image acquisition is performed using spinning disk confocal microscope (Andor, USA), ImageJ processes the image.

**Cell proliferation assays** Cell proliferation was measured by counting viable cells using the Countess II Automated Cell Counter UNIT (Life Technologies, UK). Briefly, HCT116 or HCT116 eIF4E2-KO cells hypoxia (1% O_2_) treatment or hypoxia combined with overexpression of eIF4E2 were seeded at 1×10^4^ cells per well in

6-well plates in six copies, the number of viable cells in each well was measured at 0, 1, 2, 3, 4 and 5 days.

**Immunohistochemistry** Immunohistochemistry was performed as described [[13](#_ENREF_13)]. Five-micrometer-thick paraffin sections were stained for p21 and p53. Briefly, 5 μm paraffin sections were dewaxed with xylol and hydrated through downward alcohol series. Antigen retrieval was assessed by microwaving in citrate buffer (pH 6.0). Slides were equilibrated in Tris-buffered saline (pH 7.4), quenched with H_2_O_2_, blocked with biotin/avidin, and goat serum and blocking reagent (VECTOR M.O.M. Immunodetection Kit) and incubated with primary antibodies overnight at 4°C. Corresponding biotinylated secondary antibodies were coated for 1 hour at room temperature. After washing slides were incubated with extravidin-POD conjugate and washed three times before staining with 3, 3-diaminobenzidine-tetrahydrochloride (DAB). POD oxidizes DAB producing a brown precipitate.

**Manual microinjection of DNA** The plasmid pminiTol2-5UAS-eIF4E2-Sg1180 was mixed with transposase mRNA in a ratio of 1:2. The mixture was microinjected (20 pg/nl) into 1-2 cell stage embryos of zebrafish. Plasmids were prepared in 10 mM Tris-HCl pH 8.0 and 0.25 mM EDTA, at a final concentration of 20 ng/µL and backfilled in glass injection capillaries. Individual zygotes were fixed by suction to a holding pipet, while the injection capillary was pushed though the zona pellucida and cell membrane. Approximately 10 pL plasmid solution was then injected into the cytoplasm using an Eppendorf transjector 5246 (Eppendorf, Germany).

**Synthesis of transposase mRNA** Transposase mRNA was synthesised in vitro using pCS2 which carries Tol2 transposase cDNA. The plasmid pCS2 was digested with NotI and purified by phenol: chloroform extraction and ethanol precipitation. RNA synthesis was performed using mMessage mMachine SP6 kit (Ambion). The synthesized mRNA was purified with Quick spin column.

**ELISA analysis** Mouse blood samples were collected, stewed 2 hr at room temperature or overnight at 4 °C, and then centrifuged (3000 rpm, 10 min) to gain serum. Secretion of mouse IL1β, IL6 and IL8 was measured using Mouse Interleukin-1β (IL-1β) ELISA Kit (Beyotime, PI301), Mouse IL-6 ELISA Kit (Beyotime, PI326), Mouse IL-8 ELISA Kit (MYBiosource, MBS7606860) according to the manufacturer’s instructions.

**CRISPR/Cas9 Gene Knockout of eIF4E2 in HCT116 Cells** sgRNAs targeting eIF4E2 gene were cloned into pX330-U6-Chimeric_BB-CBh-hSpCas9 (Addgene, #42230 ) as previously described [[14](#_ENREF_14)]. sgRNA sequences are as follows: CAG CCT GCC TGG CAT TCT AGtgg. HCT116 cells were cotransfected with plasmid pX330 and cas9 Nuclease (GenScript, Z03386-50) containing sgRNAs targeting exon 7 of eIF4E2-201. Single-cell clones were selected with puromycin and tested for positive by using PCR, using primers P1: GAG GTC TGC CTC TGG GAC TT /P2: CGA TGA CGC CAG CTT CAA T and ten individual clones were used for further experiments.

**CRISPR/Cas13d Gene Knockdown of eIF4E2 in liver** the plasmid expressing cas13d protein is EF1a-CasRx-2A-EGFP (Addgene, #109049) [[15](#_ENREF_15)]. sgRNAs targeting eIF4E2 gene were cloned into CasRx pre-gRNA cloning backbone (Addgene, #109054). sgRNA#1 sequences are as follows: AGAGGUUUUGAAAAAGGAGCCUU. sgRNA#2 sequences are as follows: CACAUUCAAUCGCGGCUUCCAGA. Two different sg-eIF4E2 specially targeting the eIF4E2 isoform with GSK3β binding motif were combined for improved knockdown efficiency in liver. Use hydrodynamic liver injection, plasmid DNA suspended in 2 ml saline was injected via the tail vein in 5-7 s into 8-week-old male mice. 96 hours after the plasmid injection, the mice were treated with peptide e2-I

(35 mg/kg) or not, and then the mice were sacrificed for analysis. Mouse primary hepatocytes were isolated by standard two-step collagenase perfusion and purified by 40% Percoll (Sigma) at low-speed centrifugation (1000 rpm, 10 min). Hepatocytes were resuspended in DMEM plus 10% fetal bovine serum (FBS) for FACS, and specific cell populations were used for RNA extraction or Western blot analysis.

**Peptide or drug administration and intermittent hypoxia (IH) exposure of DEN-treated mice** 14-d-old male mice were intraperitoneally injected with 6mg/kg DEN (Sigma). After 6 weeks of age, mice were injected intraperitoneally with peptide e2-I or e2-S (35mg/kg) twice a week for 6 weeks, and exposed to hypoxia (12% O_2_) for 8 hours after each peptide injection (intermittent hypoxia exposure).

For senolytics drugs treatment, after 8weeks injection, the drugs (dasatinib and quercetin) (MedChemExpress) were diluted in 100 μl of 10% PEG 400 and delivered by oral gavage once a month for three months at dosages of 5 dasatinib and 50 mg/kg quercetin body weight.

Tannic acid (TA) solution (10mM) and e2-I solution (0.5mM) were prepared [[16](#_ENREF_16)]. TA solution was diluted to 0.05, 0.1, 0.5 or 1 mM. Diluted TA solution and

e2-I solution mixed vigorously at a volumetric ratio of 1:1 corresponding to the

following stoichiometric ratios of [e2-I]/[TA]: 10, 5, 1 and 0.5. The ratio of

[e2-I]/[TA] equal to 5 is the most appropriate. After 30min incubation at room

temperature, 200ul TANNylated-e2-I solution was intravenously injected into mouse

tail veins (8-week-old male BALBc).

**References**

1. Freemantle SJ, Portland HB, Ewings K, Dmitrovsky F, DiPetrillo K, Spinella MJ, et al. Characterization and tissue-specific expression of human GSK-3-binding proteins FRAT1 and FRAT2. *Gene* **291**, 17-27 (2002).

2. Liu BH, Jobichen C, Chia CSB, Chan THM, Tang JP, Chung TXY, et al. Targeting cancer addiction for SALL4 by shifting its transcriptome with a pharmacologic peptide. *Proceedings of the National Academy of Sciences* **115**, E7119-E7128 (2018).

3. Zhang M, Zhang J, Chen X, Cho SJ, Chen X. Glycogen synthase kinase 3 promotes p53 mRNA translation via phosphorylation of RNPC1. *Genes & development* **27**, 2246-2258 (2013).

4. Moyle RL, Carvalhais LC, Pretorius LS, Nowak E, Subramaniam G, Dalton-Morgan J, et al. An Optimized Transient Dual Luciferase Assay for Quantifying MicroRNA Directed Repression of Targeted Sequences. *Front Plant Sci* **8**, 1631 (2017).

5. Kim D, Langmead B, Salzberg SL. HISAT: a fast spliced aligner with low memory requirements. *Nat Methods* **12**, 357-360 (2015).

6. Liao Y, Smyth GK, Shi W. featureCounts: an efficient general purpose program for assigning sequence reads to genomic features. *Bioinformatics* **30**, 923-930 (2014).

7. Love MI, Huber W, Anders S. Moderated estimation of fold change and dispersion for RNA-seq data with DESeq2. *Genome Biol* **15**, 550 (2014).

8. Subramanian A, Tamayo P, Mootha VK, Mukherjee S, Ebert BL, Gillette MA, et al. Gene set enrichment analysis: a knowledge-based approach for interpreting genome-wide expression profiles. *Proceedings of the National Academy of Sciences of the United States of America* **102**, 15545-15550 (2005).

9. Zhang H, Alsaleh G, Feltham J, Sun Y, Napolitano G, Riffelmacher T, et al. Polyamines Control eIF5A Hypusination, TFEB Translation, and Autophagy to Reverse B Cell Senescence. *Molecular Cell* **76**, 110-125.e119 (2019).

10. McMahon C, Baier AS, Pascolutti R, Wegrecki M, Zheng S, Ong JX, et al. Yeast surface display platform for rapid discovery of conformationally selective nanobodies. *Nature structural & molecular biology* **25**, 289-296 (2018).

11. Chung C-I, Zhang Q, Shu X. Dynamic Imaging of Small Molecule Induced Protein–Protein Interactions in Living Cells with a Fluorophore Phase Transition Based Approach. *Analytical chemistry* **90**, 14287-14293 (2018).

12. Zhang Q, Huang H, Zhang L, Wu R, Chung CI, Zhang SQ, et al. Visualizing Dynamics of Cell Signaling In Vivo with a Phase Separation-Based Kinase Reporter. *Molecular cell* **69**, 334-346 e334 (2018).

13. Ueberham E, Bottger J, Ueberham U, Grosche J, Gebhardt R. Response of sinusoidal mouse liver cells to choline-deficient ethionine-supplemented diet. *Comparative hepatology* **9**, 8 (2010).

14. Ran FA, Hsu PD, Wright J, Agarwala V, Scott DA, Zhang F. Genome engineering using the CRISPR-Cas9 system. *Nature Protocols* **8**, 2281-2308 (2013).

15. Konermann S, Lotfy P, Brideau NJ, Oki J, Shokhirev MN, Hsu PD. Transcriptome Engineering with RNA-Targeting Type VI-D CRISPR Effectors. *Cell* **173**, 665-676 e614 (2018).

16. Shin M, Lee HA, Lee M, Shin Y, Song JJ, Kang SW, et al. Targeting protein and peptide therapeutics to the heart via tannic acid modification. *Nat Biomed Eng* **2**, 304-317 (2018).

**Supplementary figure legends**

**Supplementary Fig. 1** **eIF4E2 regulates GSK3β proline-directed kinase activity.** Related to **Figure 1**

**A, B** RBM38 directly interacts with eIF4E2. GST-agarose beads bound GST-RBM38 or GST proteins were incubated with purified His-eIF4E2 (A), or GST-eIF4E2 with His-RBM38 (B) The elution was analyzed by WB using antibodies as indicated. **C, D** Endogenous eIF4E2 and RBM38 interaction. Cell extracts were subjected to

co-immunoprecipitation (Co-IP) with anti-RBM38 antibody (C), anti-eIF4E2 antibody (D), or IgG, followed by WB with indicated antibodies. **E** Phosphospecific antibodies are made against phosphorylated synthetic peptides corresponding to an amino acid sequence that contains RBM38-Ser195. **F, G** eIF4E2 directly interacts with GSK3β. The experiments were done as described in (A). **H, I** Endogenous GSK3β and eIF4E2 interaction. The experiments were done as described in (C), except that the indicated antibodies were used. **J** e2-I inhibits eIF4E2-GSK3β binding. The purified GST-eIF4E2 was incubated with different concentrations of e2-I (0,100, 500, 1000 pmol) for 1 hour, then incubated with purified His-GSK3β, followed by WB. **K** e2-S has no effect on the phosphorylation of RBM38-Ser195 at 5, 10, and 20 μM for 24 and 48-hours treatment. **L** Identifying the eIF4E2 binding motif of GSK3β. eIF4E2 directly interacts with, but not with GSK3β (Δ314-329). **M** G3-I inhibits the interaction of eIF4E2 and GSK3β. The experiments were done as described in (J), except that G3-I was used. **N** Overrepresented phosphorylation motifs. Phosphorylation motifs were extracted by using Motif-X algorithm for phosphoproteomics data and threshold for significance was set to P< 0.000001. **O** eIF4E2 further increases the level of the phosphorylation of HIF1α-Ser589 in the presence of GSK3β in vitro kinase assay. The recombinant proteins Flag-GSK3β, His-eIF4E2 were purified and incubated with His-HIF1α or His-HIF1α(S589A), as indicated, in the kinase reaction mixture, followed by WB. **P** Multiple-sequence alignment of c-termini of human eIF4E2, including isoform A-G with or without GSK3β binding motif. **Q** eIF4E2-KO HCT116 were generated by CRISPR/Cas9 technology. The used gRNA was indicated that specially targeting eIF4E2 isoforms with GSK3β binding motif. The CRISPR targeted locus was PCR amplified from genomic DNA using primers P1/P2 and PCR products were size-separated by electrophoresis on a 2% agarose gel.

**Supplementary Fig. 2 eIF4E2**-**GSK3β maintains p53 phosphorylation at multiple S/T-P sites.** Related to **Figure 2**

**A** e2-I inhibits p53 protein translation. Cells were treated with 5μM e2-I or scrambled e2-S for 24 hours. After cells were exposed to L-azidohomoalaine (AHA), cell lysates were incubated with the reaction buffer containing biotin/alkyne reagent. The biotin-alkyne-azide-modified protein complex was pulled down and analyzed by WB (left panel). The cell lysates before pull-down (input) were analyzed by WB (right panel). **B** e2-I extends the half-life of p53 protein. Cells were treated with e2-I, then treated with or without cycloheximide (CHX, 0.1mg/mL) for up to 45min, followed by WB. **C** Four S–P sites (Ser33–Pro, Ser46–Pro, Ser127–Pro, Ser315–Pro) and two T-P sites (Thr81–Pro and Thr150–Pro) of human p53 protein. **D** G3-I inhibits the CPT-induced p53 phosphorylation at multi-S-P. Cells were treated with 5μM G3-I or scrambled peptide, along with mock-treated or treated with 200 nM CPT for 24 hours, followed by WB with indicated antibodies. **E** G3-I inhibits the nocodazole-induced phosphorylation of p53 Thr81. The experiment was done as (D), except that 50ng/ml Nocodazole were used for 24 hours. **F** G3-I inhibits multi-S/T-P phosphorylation of p53 at basal conditions.

**Supplementary Fig. 3 Dephosphorylated p53 promotes senescence by repressing transcription.** Related to **Figure 3**

**A** SA-β-Gal staining of A549 and H1299 cells treated with 5μM e2-I or e2-S for 96 hours (upper panel), quantitative analysis percentages of SA-β-Gal positive cells (right panel n=3). Followed by WB with indicated antibodies (lower panel). Scale bars, 25 μm. **B** e2-I reduces the size and volume of xenografts depending on p53. Cells were treated with 5μM e2-I or scrambled e2-S for 24 hours, then were injected subcutaneously into the nude mice for 14 days. Representative images of xenograft are shown. **C** Tumors weight was measured and shown in the scatter plot (n=4). **D** Gene set enrichment analysis (GSEA) plot showed e2-I treatment correlated with neurodegerative disease, including Alzheimer's disease (left panel), Huntington's disease (middle panel), Parkinson’s disease (right panel). **E** GSEA of differential expression genes upon e2-I treatment and GSEA plot showed e2-I inhibits WNT signaling pathway. NES, normalized enrichment score; NOM P, normalized p-value; FDR, false discovery rate. **F** Mutant p53-6A suppresses the expression of TRX1. Vectors expressing FLAG tagged p53, p53-6A or p53-6D, were mock-transfected or transfected into p53-null HCT116 cells for 48 hours, followed by WB with indicated antibodies.

**Supplementary Fig. 4 Dephosphorylated p53 promotes senescence by repressing transcription.** Related to **Figure 3**

**A, B** peptide e2-I or p53-6A inhibit the mRNA expression of HNRNPD, EEF2, GAMT, NEK9 by RT-PCR analysis. Densitometric analysis of the bands was performed using ImageJ software, the ratio of genes/β-actin for e2-I, p53-6A treatment was quantified (n=3). **C**, e2-I downregulates the expression of TOPBP1, TRX1, BCL-2 or BAD depending on p53. HCT116 or HCT116-p53 null cells were treated with 5μM e2-I or scrambled e2-S for 24 hours and analyzed by WB with indicated antibodies. **D, E** A schematic representation of human TOPBP1(D) and TRX1(E) promoter with the predicted p53 responsive elements (REs). The insert shows the sequence of RE, as well as its localization (upstream of the ATG). The locations of PCR primers used in FLAG-CHIP assays are shown. **F, G** p53 associates with TOPBP1or TRX1 promoter. FLAG-CHIP assay was performed in p53-null HCT116 cells, which were mock-transfected or transfected with vectors expressing FLAG-tagged p53, p53-6A or p53-6D, for 36 hours. The cell lysates were immunoprecipitated with anti-FLAG antibodies and analyzed by RT-PCR. p53 binding to TOPBP1 and TRX1 promoters was estimated by RT-PCR and represented as fold difference from the value measured in control-transfected (n=3). p21 as a positive control, GAPDH as a negative control.

**Supplementary Fig. 5 Dephosphorylated p53 promotes senescence by repressing transcription.** Related to **Figure 3**

**A** p53 directly inhibits TOPBP1 transcription activity. p53-null HCT116 cells were transfected with luciferase reporter constructs as indicated, along with mock-transfected or transfected with vectors expressing p53, p53-6A or p53-6D. Luciferase activity in cell lysates was measured and normalized by renilla activity using a dual-luciferase assay system. All data represent at least three independent experiments with similar results. p value <0.001 (***) vs control. **B** Schematic representation of TOPBP1 promoter with REs, as well as the luciferase reporter gene constructs. pGL3-TOPBP1 contains p53 REs and pGL3-TOPBP1(ΔREs) lacking p53 REs as indicated. **C** p53 directly inhibits TRX1 transcription activity. The experiments were done as described in (A). **D** Schematic representation of TRX1 promoter, as well as the luciferase reporter gene constructs. **E** GST pull-down assay demonstrated that Nanobody Nb-28A1 directly interacts with eIF4E2, but not interact with eIF4E2 (Δ231-242) or Frat. **F** Nanobody Nb-28A1 interacts with endogenous eIF4E2. Vector expressing HA-tagged Nb-BV025 or HA-tagged Nb-28A1 was transfected into HCT116 cells for 48 hours. The cell lysates were immunoprecipitated with anti-HA beads and analyzed by WB. **G** Nanobody Nb-28A1 inhibits the interaction of eIF4E2 and GSK3β. The purified GST-eIF4E2 was incubated with different concentrations of Nb-28A1 (0,100, 500, 1000 pmol) for 1 hour, then incubated with purified His-GSK3β, followed by WB. **H** Nanobody Nb-28A1 inhibit the mRNA expression of HNRNPD, EEF2, GAMT, NEK9 by RT-PCR analysis. Densitometric analysis of the bands was performed using ImageJ software, the ratio of genes/β-actin for Nanobody Nb-28A1 treatment was quantified (n=3).

**Supplementary Fig. 6 Hypoxia inhibits the eIF4E2**-**GSK3β pathway.** Related to **Figure 4**

**A** Hypoxia inhibits the phosphorylation of RBM38-Ser195. HCT116 cells exposure to hypoxia (1% O_2_) for 18, 24 hours, or normoxia for 24 hours, followed by WB. **B** SPPIER assay showed hypoxia inhibits eIF4E2-GSK3β interaction. Cells transiently expressed EGFP-eIF4E2-HOTag3-T2A-GSK3β-HOTag6, and then exposure to hypoxia (1% O_2_) for 2 hours. Scale bars, 1 μm. **C** L-NAME partially restores RBM38-Ser195 phosphorylation under hypoxia. Cells were mock-treated or treated with 100μM L-NAME under normoxia or hypoxia (1% O_2_) for 24 hours and subjected to WB. **D, E** Proliferation curves of cells cultured from 1 to 5 days. eIF4E2-KO HCT116 (D) or HCT116 (E) cells with or without eIF4E2 isoform A expression were incubated under normoxia or hypoxia (1% O_2_) conditions, and cell number were counted daily from days 1 to 5 (n=3). After 5 days, the cells were stained with crystal violet. The data were represented as mean ± S.D. of three independent experiments.

**Supplementary Fig. 7 Blocking eIF4E2**-**GSK3β interaction promotes liver senescence under hypoxia.** Related to **Figure 5**

**A** Ambulation counts of mice. Mice in the open field chamber over 5 min. After consecutive intraperitoneal injection of 35mg/kg e2-I or scrambled e2-S every day for 2 days, mice were housed at normoxia or physiological hypoxia (12% O_2_) conditions and tracks of mice were records at 3 or 6 days respectively (n=3). **B** Rearing counts of mice in experiment (A) Significance was evaluated with the t-test and repeated measures ANOVA. **P < 0.01. **C** Equal amounts of liver tissues extracts from experiment (A) were subjected to WB with indicated antibodies. **D** Immunohistochemical staining of liver tissue from experiment (A) for expression of p21 and p53. Scale bars, 50 μm. **E** Expression of TOPBP1 rescued e2-I induced

senescence. AAV8-TOPBP1 (1×10^11^viral genomes in 100μl saline) were intravenous injected. 2 weeks later, mice were treated with peptide e2-I as experiment (A), and mice were housed at normoxia or physiological hypoxia (12% O_2_) conditions. 5 days later, SA-β-Gal staining of liver were sectioned and then counterstained with nuclear fast red (upper panel). Scale bars, 25 μm. Equal amounts of liver tissue were subjected to WB for checking the expression of TOPBP1 (lower panel). **F** Expression of TRX1 rescued G3-I induced senescence. The experiments were done as described in (E), except for peptide G3-I was used. Liver tissue were stained for SA-β-Gal activity, then section and counterstained with nuclear fast red. Scale bars, 25 μm (upper panel). Equal amounts of liver tissue were subjected to WB checking the expression of TRX1 (lower panel).

**Supplementary Fig. 8 Blocking eIF4E2**-**GSK3β interaction promotes liver senescence under hypoxia.** Related to **Figure 5**

**A** Mice (6weeks old) were injected intraperitoneally with 35mg/kg e2-I or scrambled e2-S twice a week for 6 weeks. After each injection, the mice were exposed to physiological hypoxia (12% O_2_) conditions for 8 hours. SA-β-Gal staining of liver tissue blocks were performed. **B** The SA-β-Gal-stained liver tissues shown in (A) were sectioned and then counterstained with nuclear fast red (left panel), and SA-β-Gal staining results were quantified by the ratio of SA-β-Gal^+^ area to the total image area for three fields (n=3, right panel). Scale bars, 25 μm. **C, D** Scheme of G3-I/IH or synolytics drug (dasatinib and quercetin) administration of DEN-treated mice. Representative macroscopic photographs of livers (n=6). Arrowheads indicate hepatocellular carcinoma (HCCs). Scale bar: 1 cm (C). Number of surface tumours and LW/BW ratios in mice liver at 24 weeks and shown in the scatter plot (n= 6) (J). **E** Gross liver appearances of DEN-treated mice with administration of scramble peptide, e2-I or G3-I under normoxia conditions (n=5). Scale bar: 1 cm.
