# Supplementary figures and images for: Mammalian eIF4E2-GSK3β maintains basal phosphorylation of p53 to resist senescence under hypoxia
Source: Cell Death Dis. 2022 May 14;13(5):459. doi: 10.1038/s41419-022-04897-4 (PMC9107480; doi:10.1038/s41419-022-04897-4)

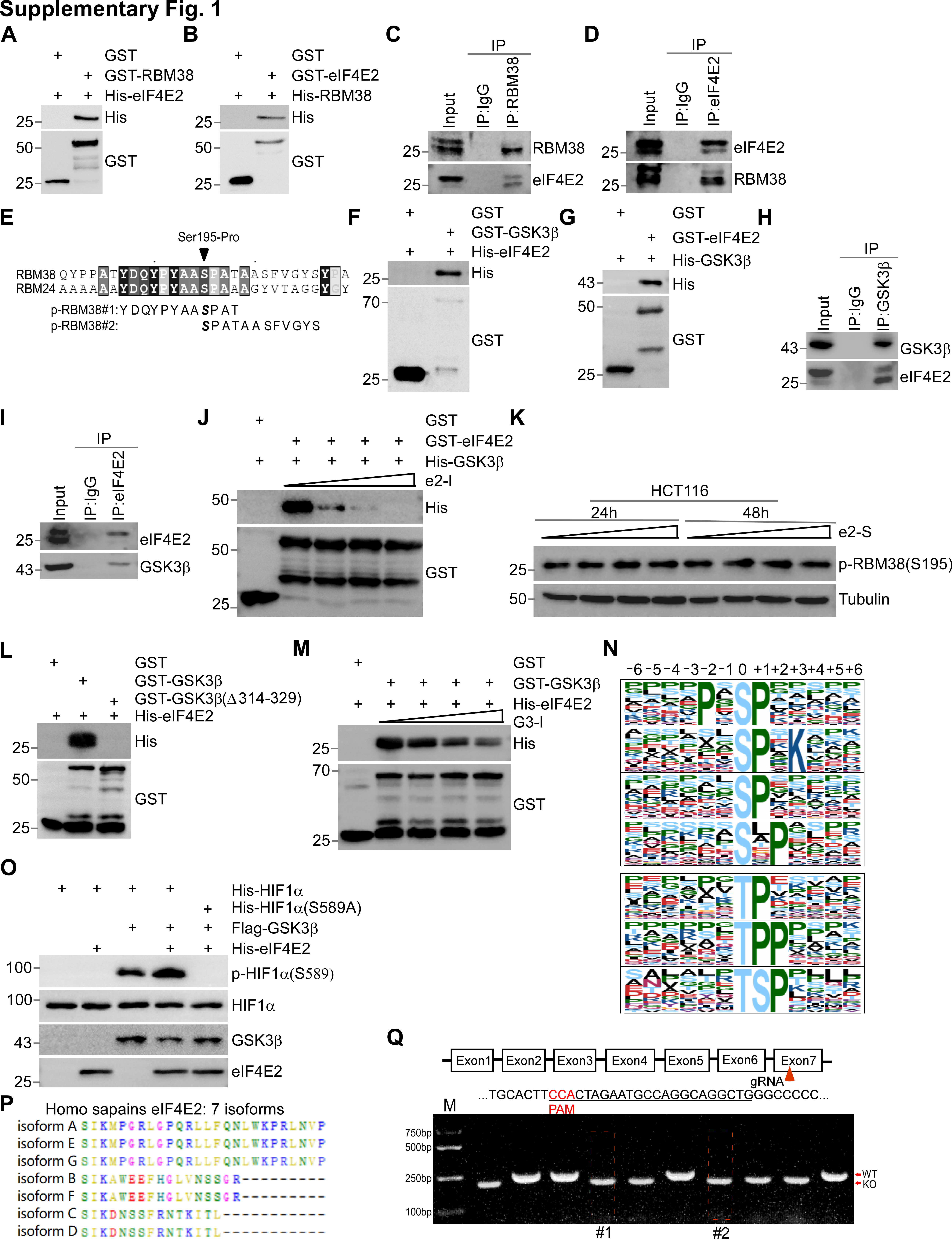

Supplement: Supplementary file 3 — Supplementary Figure 1 [file 41419_2022_4897_MOESM3_ESM.tif]

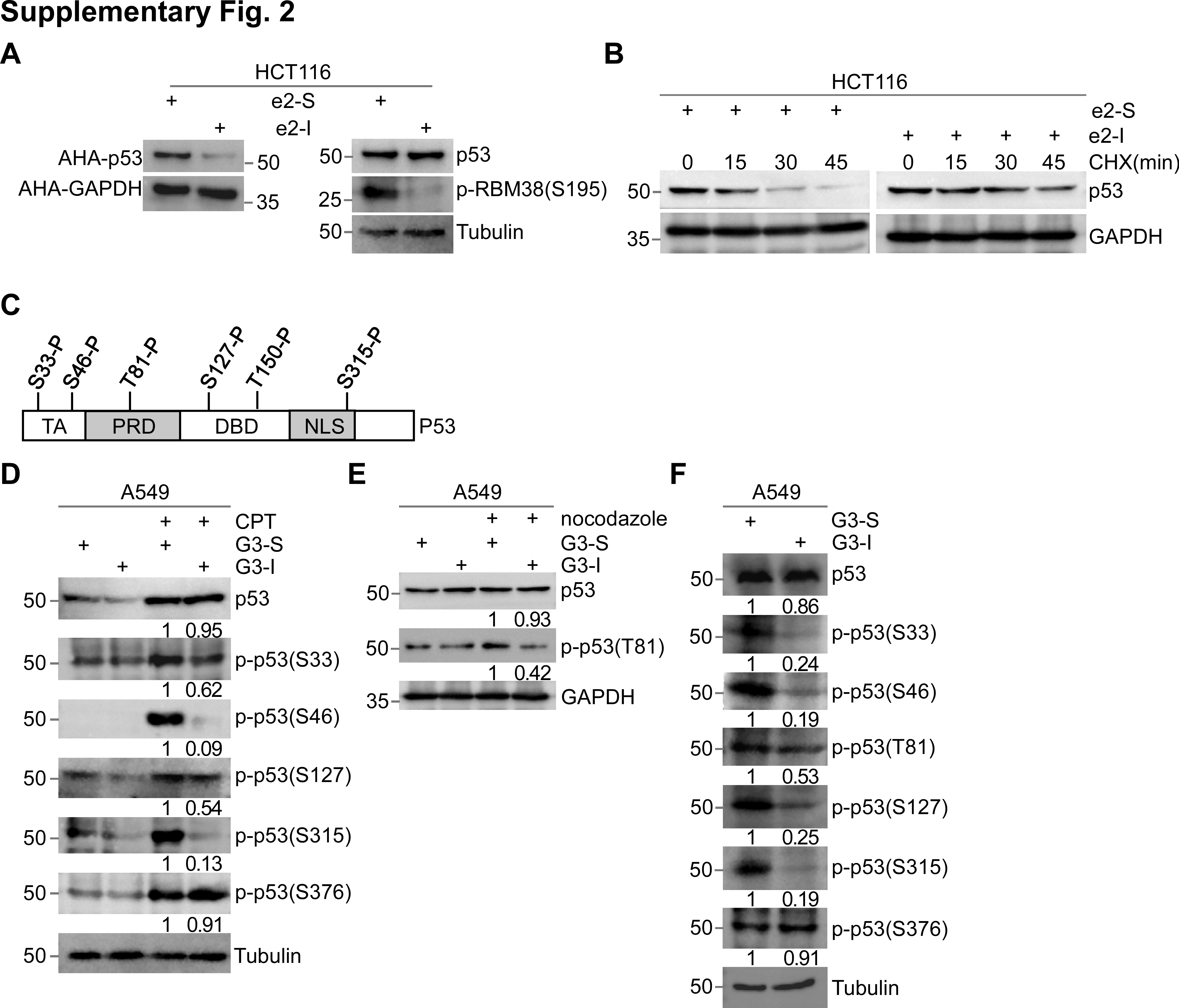

Supplement: Supplementary file 4 — Supplementary Figure 2 [file 41419_2022_4897_MOESM4_ESM.tif]

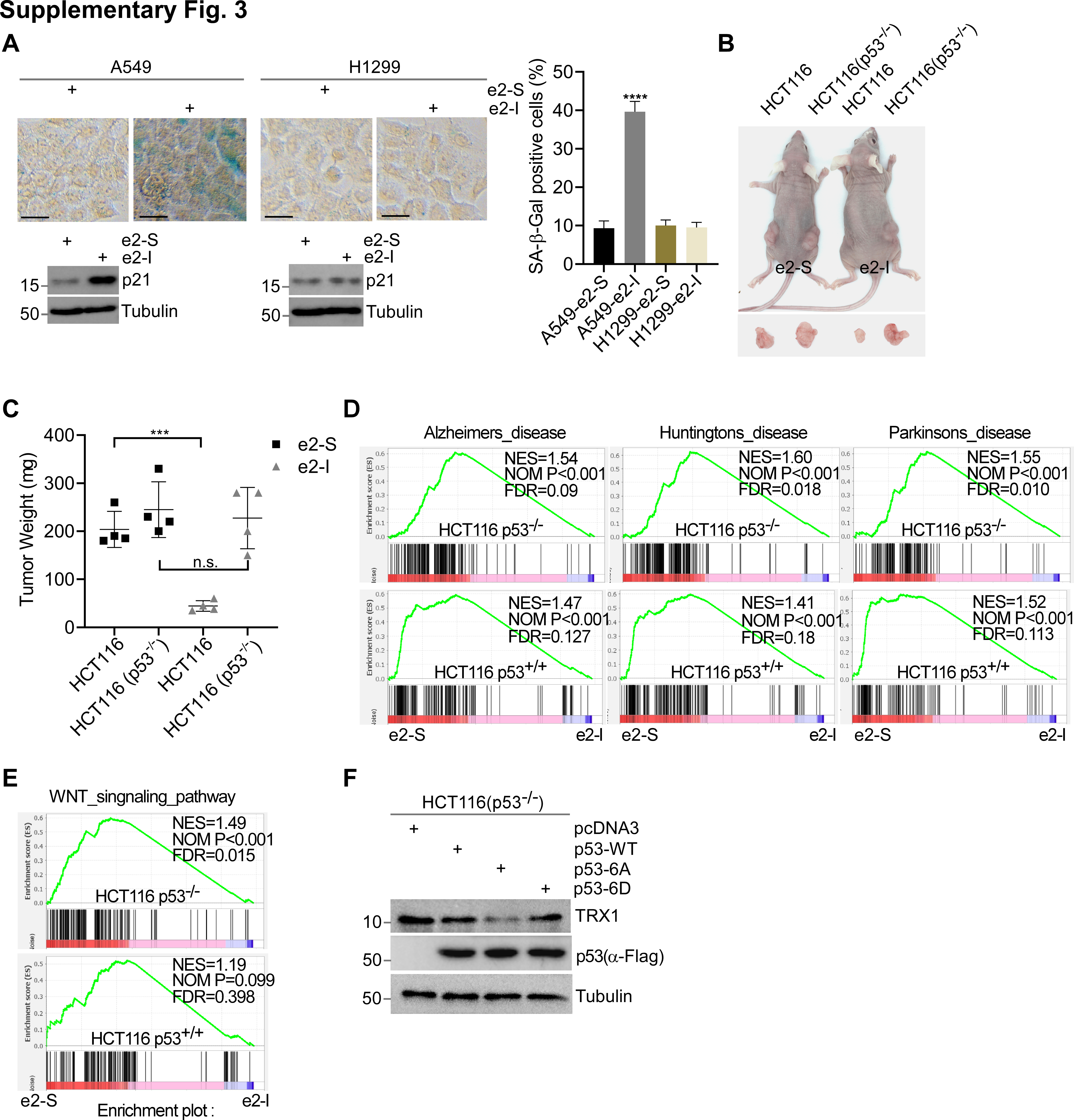

Supplement: Supplementary file 5 — Supplementary Figure 3 [file 41419_2022_4897_MOESM5_ESM.tif]

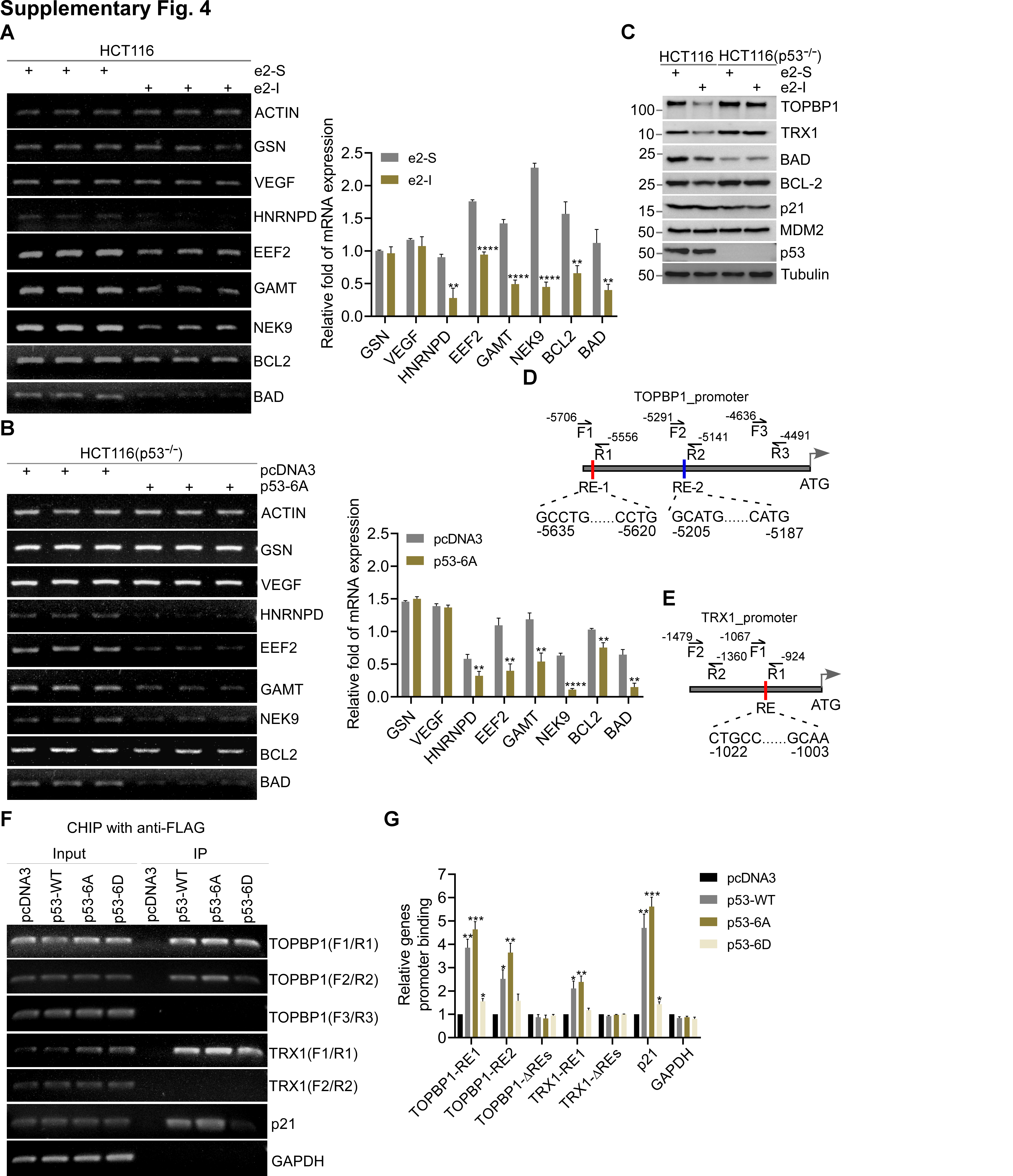

Supplement: Supplementary file 6 — Supplementary Figure 4 [file 41419_2022_4897_MOESM6_ESM.tif]

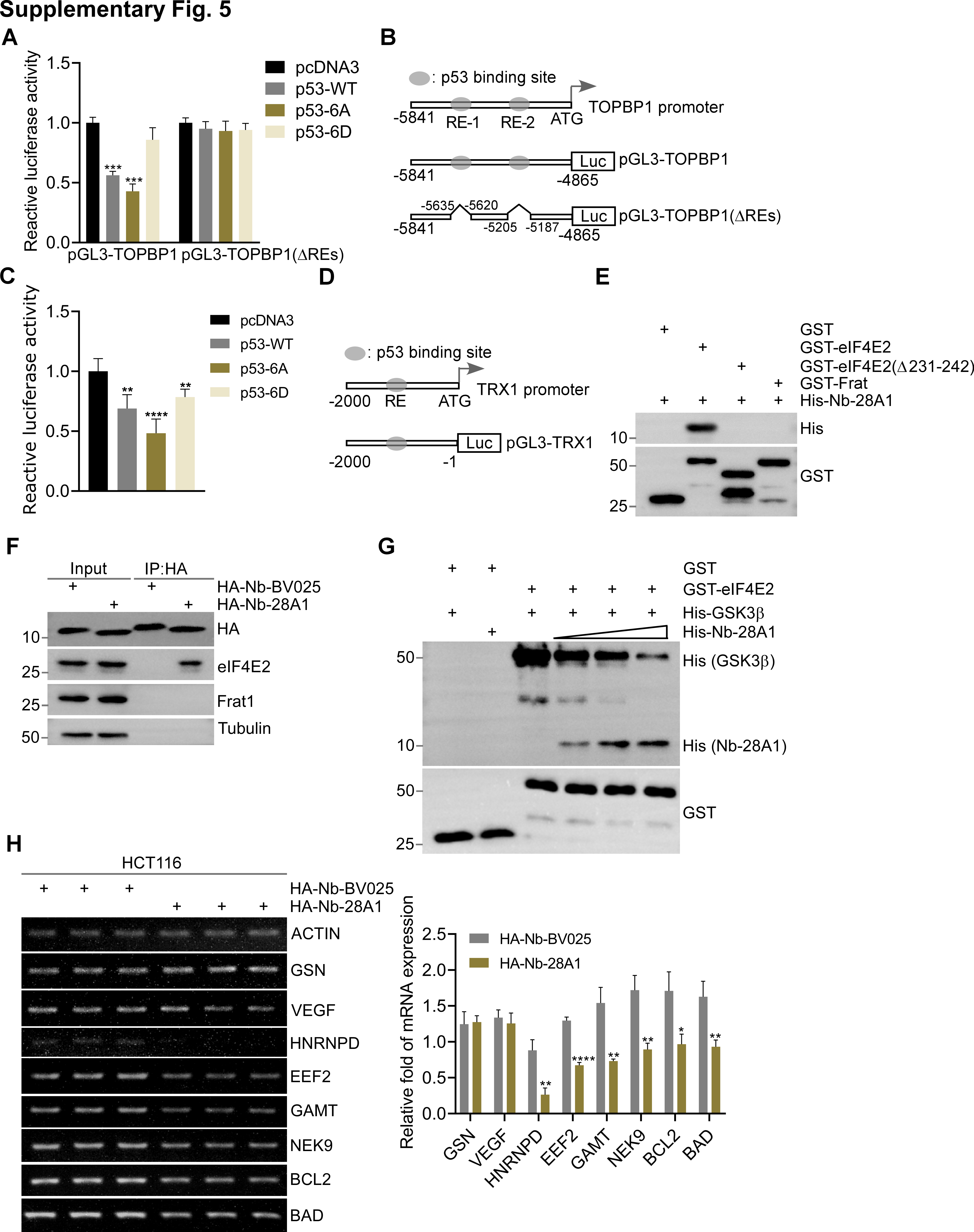

Supplement: Supplementary file 7 — Supplementary Figure 5 [file 41419_2022_4897_MOESM7_ESM.tif]

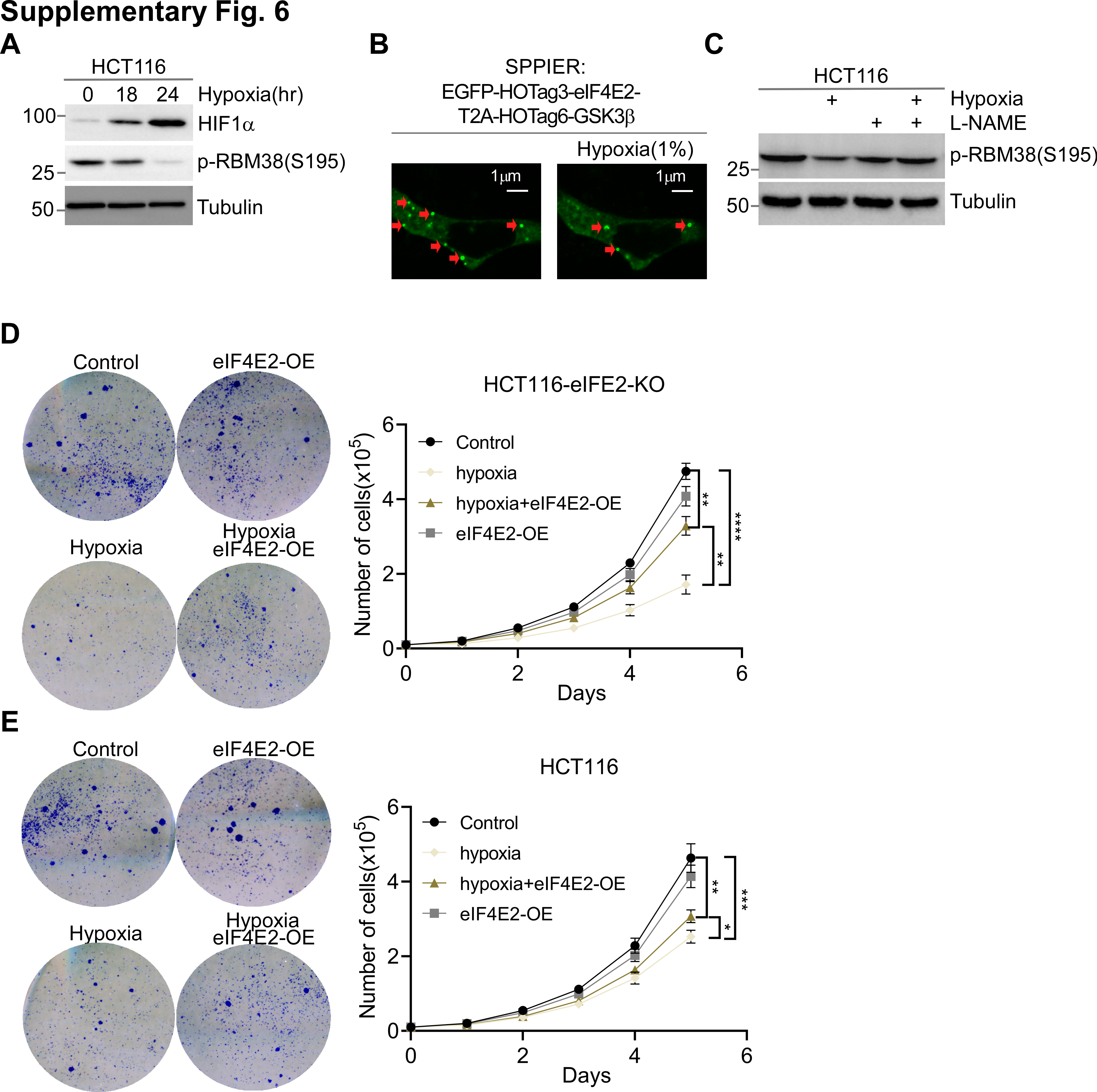

Supplement: Supplementary file 8 — Supplementary Figure 6 [file 41419_2022_4897_MOESM8_ESM.tif]

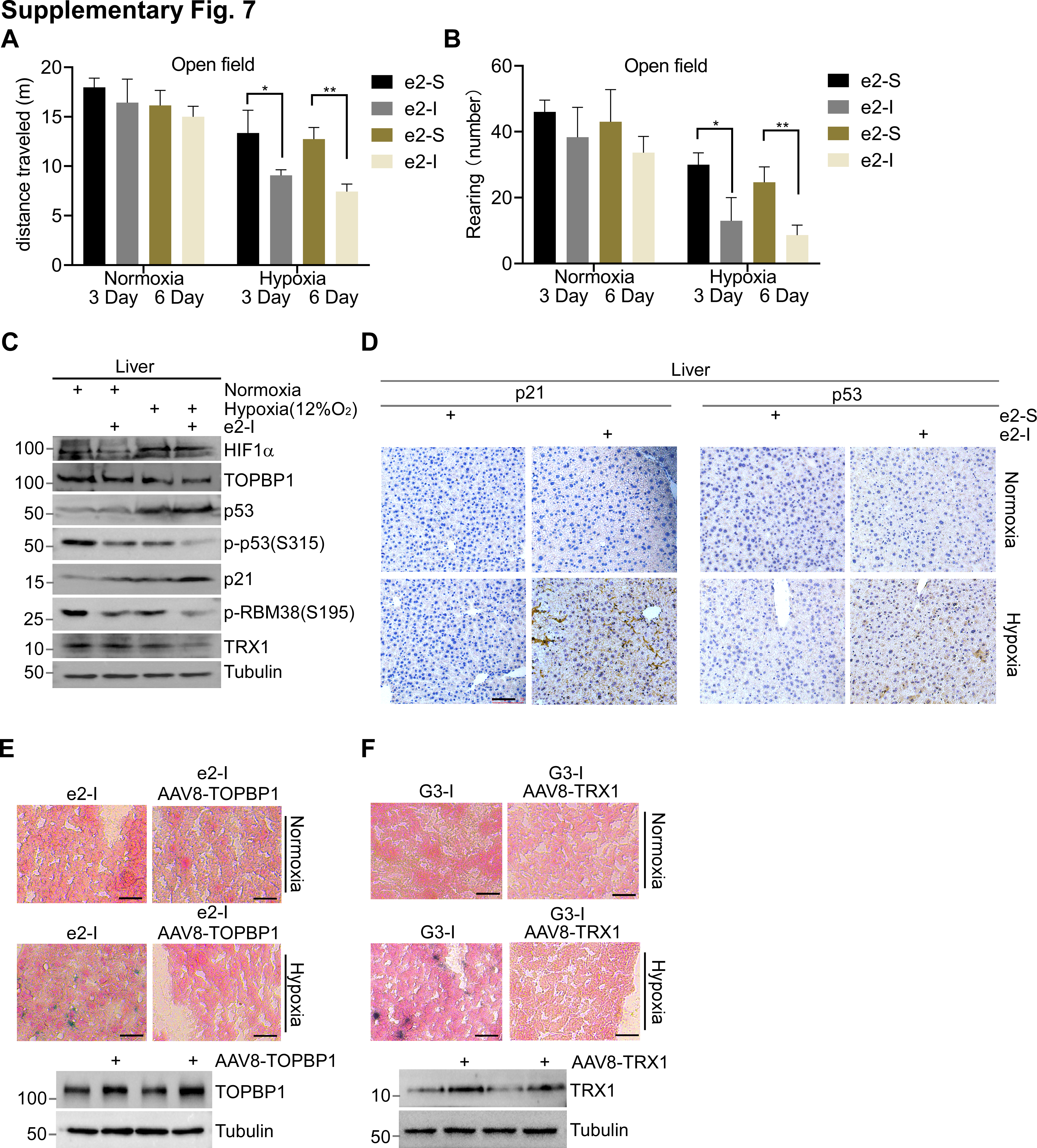

Supplement: Supplementary file 9 — Supplementary Figure 7 [file 41419_2022_4897_MOESM9_ESM.tif]

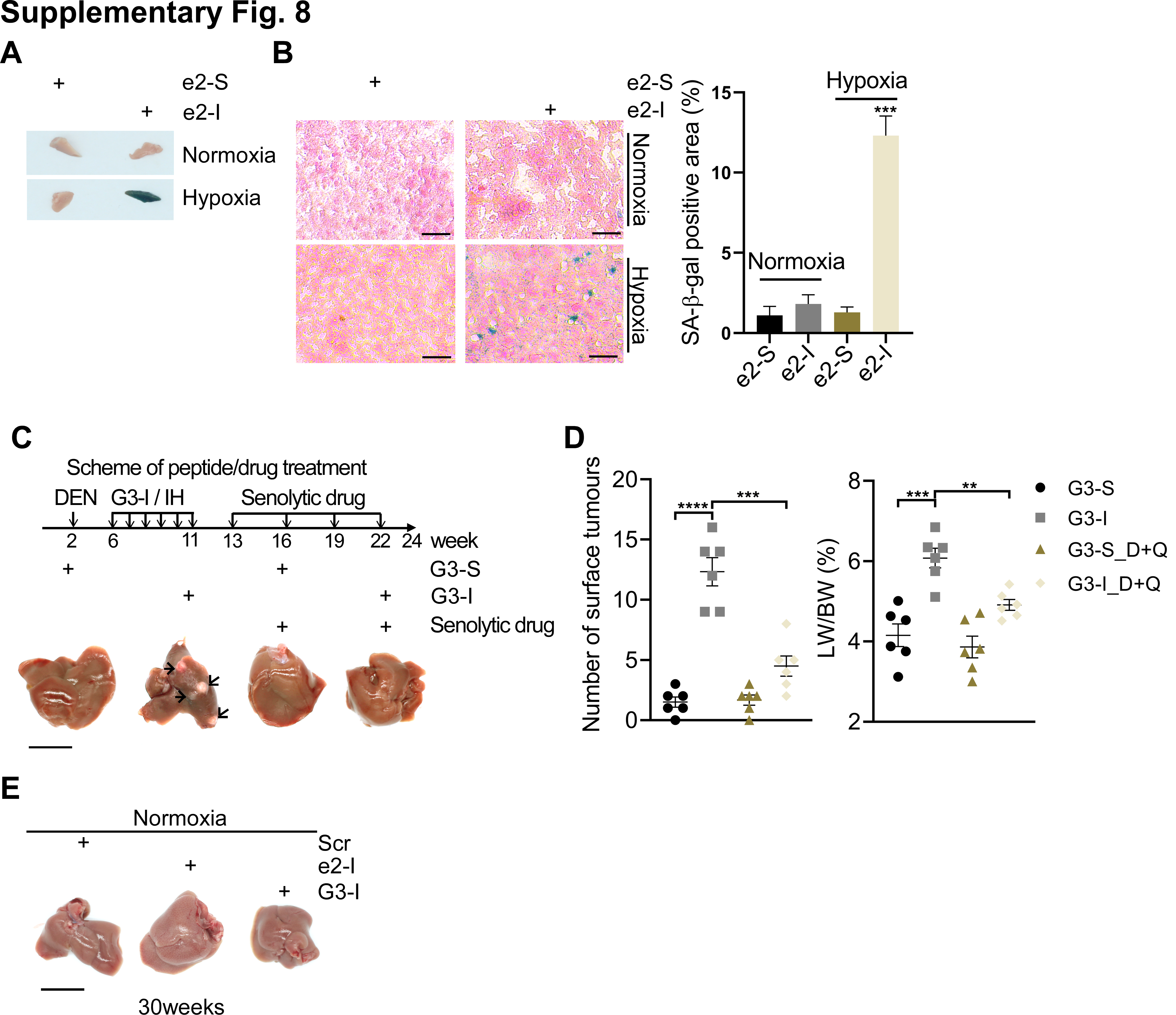

Supplement: Supplementary file 10 — Supplementary Figure 8 [file 41419_2022_4897_MOESM10_ESM.tif]
